# Development of a synthetic gene network to modulate gene expression by mechanical forces

**DOI:** 10.1038/srep29643

**Published:** 2016-07-12

**Authors:** Zoltán Kis, Tania Rodin, Asma Zafar, Zhangxing Lai, Grace Freke, Oliver Fleck, Armando Del Rio Hernandez, Leila Towhidi, Ryan M. Pedrigi, Takayuki Homma, Rob Krams

**Affiliations:** 1 Department of Bioengineering, Imperial College London, Exhibition Road, London, SW7 2AZ, United Kingdom; 2 Government College University, GC University, Katchery Road, Lahore, 54000, Pakistan; 3 Department of Mechanical Engineering, National University of Singapore, 9 Engineering Drive 1, 117575, Singapore; 4 Department of Biomedical Engineering, National University of Singapore, 4 Engineering Drive 3, 117583, Singapore; 5 Institute of Child Health, University College London, 30 Guilford Street, London, WC1N 1EH, United Kingdom; 6 Department of Engineering, University of Cambridge, Trumpington Street, Cambridge, CB2 1PZ, United Kingdom; 7 Institute for Integrated Cell-Material Sciences, Kyoto University, Yoshida Ushinomiya-cho, Sakyo-ku, Kyoto, 606-8501, Japan

## Abstract

The majority of (mammalian) cells in our body are sensitive to mechanical forces, but little work has been done to develop assays to monitor mechanosensor activity. Furthermore, it is currently impossible to use mechanosensor activity to drive gene expression. To address these needs, we developed the first mammalian mechanosensitive synthetic gene network to monitor endothelial cell shear stress levels and directly modulate expression of an atheroprotective transcription factor by shear stress. The technique is highly modular, easily scalable and allows graded control of gene expression by mechanical stimuli in hard-to-transfect mammalian cells. We call this new approach mechanosyngenetics. To insert the gene network into a high proportion of cells, a hybrid transfection procedure was developed that involves electroporation, plasmids replication in mammalian cells, mammalian antibiotic selection, a second electroporation and gene network activation. This procedure takes 1 week and yielded over 60% of cells with a functional gene network. To test gene network functionality, we developed a flow setup that exposes cells to linearly increasing shear stress along the length of the flow channel floor. Activation of the gene network varied logarithmically as a function of shear stress magnitude.

Mechanotransduction, the ability of cells to sense, process and respond to mechanical forces, is increasingly recognised to play a crucial role in a variety of physiological processes including tissue formation and regeneration, angiogenesis, hearing and sensing^1^,^2^,^3^. In addition to physiological processes, mechanical forces have been shown to play an important role in diseases such as osteoarthritis, atherosclerosis, tumour growth and metastasis^4^,^5^,^6^,^7^,^8^,^9^,^10^,^11^,^12^,^13^,^14^,^15^. Mechanotransduction starts with the sensing of mechanical force by a group of cell membrane molecules, collectively known as mechanosensors^7^. These mechanosensitive molecules include G-protein coupled receptors (G-PCRs), ion channels and receptor tyrosine kinases^16^. Following sensing, the mechanical signal is converted into biochemical signals inside the cell, which then activate a cascade of signalling events, ultimately leading to gene expression up- or down-regulation. For example, in the case of endothelial shear stress sensing, it is estimated that 1000–2000 genes are involved in the mechanotransduction process, regulating 8–10 transcription factors^15^,^17^,^18^,^19^,^20^,^21^.

Despite the emerging importance of the mechanotransduction field, specific interventions to modulate mechanotransduction are currently not available, substantially hampering progress towards treatment of the above mentioned mechanosensitivity-related diseases. Thus, there is a need to investigate how varying levels of a particular mechanical stimulus affect the degree of activation or deactivation of signaling molecules within mechanotransduction signaling pathways. Additionally, it is of crucial importance to be able to modulate gene expression (e.g., therapeutic genes and transcription factors) by mechanical input signals in cells exposed to particular (pathological) mechanical environments.

To address these needs, we developed a novel synthetic gene network to monitor shear stress sensing activity and regulate gene expression in a graded manner in mammalian cells. The network was inserted into cells via a bespoke hybrid transfection procedure that overcame difficulties in inserting large DNA constructs into hard-to-transfect mammalian cells. Once transfected, cells were seeded into a custom flow chamber that imposed a range of linearly increasing shear stress over the length of the chamber, which demonstrated increased activation of the transfected gene network as measured by GFP signal with increased shear stress. Future work will employ this modular network to monitor pharmaceutical targets of specific mechanotransduction molecules to attenuate pathological mechanical signaling.

## Results and Discussion

### Design of the mechanosensitive gene network

The tremendous importance of G-PCRs in pharmacology and the extensive characterization of their mechanosensitive nature^22^,^23^, led us to build our shear sensitive gene network around a G-PCR. We first determined the feasibility of using the G-PCR by performing a comparative structural bioinformatics analysis of 6 known mechanosensitive G-PCRs: BDKRB2, FPR1, PTHR1, S1PR1, GPER1 and DR5 (Fig. S1). Using information from several database’s (UniProt, NCBI Protein and RCSB PDB) as well as bioinformatics analysis tools (Clustal Omega, TMHMM, Phobius, and Phyre), we concluded that alteration of the N- or C-terminus of the G-PCR should not affect its mechanosensitivity as the identified residues were distributed throughout the trans-membrane helices (Fig. S1).

**Figure 1:**
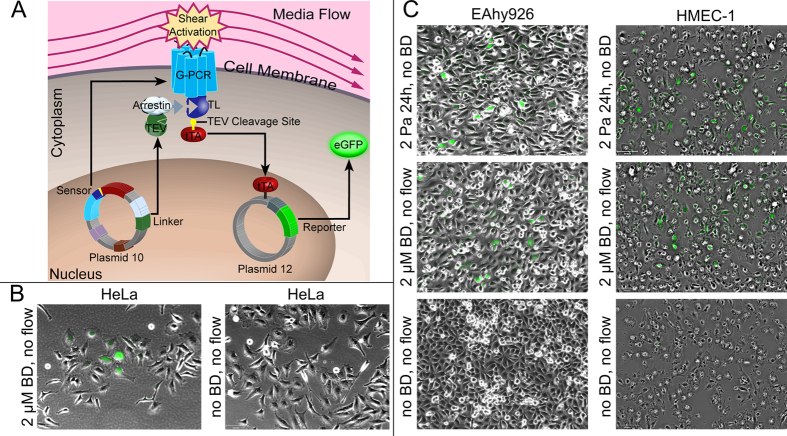
Design of the mechanosensitive synthetic gene network and its validation in HeLa and endothelial EA.hy926 and HMEC-1 cells. (**A**) Illustration of salient network components. Shear stress induction of the G-PCR causes Arrestin binding to the GPCR tail domain (TL), leading to cleavage of the TEV cleavage site linker by TEV. Released tTA promotes transcription of the eGFP reporter gene. (**B**) The gene network is functional in HeLa cells. Superimposed green fluorescent and phase contrast microscopy images of HeLa cells transfected with the gene network under static (i.e. no flow) conditions. HeLa cells were electroporated with the gene network and were either treated with 2 μM final bradykinin concentrations or were left untreated. Bradykinin treatment yielded ≈10% green fluorescent HeLa cells. N = 3, S.D.≈10%, 10X magnification. (**C**) The gene network is functional in endothelial EA.hy926 and HMEC-1 cells. Superimposed green fluorescent and phase contrast microscopy images of EA.hy926 and HMEC-1 endothelial cells transfected with the gene network. Cells were electroporated with the gene network and subjected either to 2 Pa shear stress for 24 hours, or to 2 μM bradykinin under static conditions, or left untreated under static conditions. Bradykinin treatment and flow exposure yielded ≈10% green fluorescent EA.hy926 and HMEC-1 cells. N = 3, S.D.≈10%, 10X magnification.

We designed our synthetic gene network to assay the mechanosensitive G-PCR activity by extensive modification of an existing assay^24^ (Fig. S2). Our mechanosensitive network consists of a sensor module, a reporter module, and a linker module that signals between the sensor and reporter (Fig. 1A and supplemental methods). The sensor module is composed of the Bradykinin B2 G-PCR^23^ linked to the tetracycline-controlled transcriptional trans-activator (tTA), with the linker consisting of a 7 amino acid long Tobacco etch virus (TEV) protease cleavage site. The reporter module contains eGFP under the control of a tTA dependent promoter. β-Arrestin2 signalling protein fused to the TEV protease enzyme constitutes the linker module. Upon activation of the G-PCR by shear stress or by an extracellular ligand, β-Arrestin2 binds to the cytoplasmic domain of the G-PCR leading to cleavage of the linker between tTA and the G-PCR by the TEV protease. Consequently, the released tTA enters the cell nucleus and promotes transcription from the reporter module. The gene network can be controlled at the shear stress sensor level by activating and inhibiting the Bradykinin B2 G-PCR, using bradykinin^25^,^26^,^27^ and HOE 140^28^,^29^,^30^,^31^ ligands, respectively. Additionally, the gene network can be inhibited at the transcription level using doxycycline^32^,^33^ (Fig. S3). After the gene network was constructed at the DNA level, it was inserted into mammalian cells to validate its functionality (Fig. 1B,C). It was also confirmed that the GFP is expressed in response to shear stress only when all components of the gene network are present, see Fig. S4 for details.

### Transfection procedure development

For transfection, electroporation was employed because it is fast (compared to setting up viral infection methods), versatile (works on a wide range of cell types), and reportedly yields high transfection efficiencies for a variety of hard-to-transfect cell types^34^,^35^,^36^. The synthetic network was first electroporated into (easy-to-transfect) HeLa cells and activated with 2 μM final concentration of Bradykinin, which demonstrated functionality of the gene network as ~10% of the cells expressed the fluorescent eGFP reporter gene (Fig. 1B). The network was further tested in (hard-to-transfect) EA.hy926 and HMEC-1 endothelial cells by 2 μM Bradykinin or 15–20 dyne/cm^2^ shear stress for 24 hours. Both stimuli resulted in ~10% cell fluorescence, which demonstrated that the network was equally sensitive to shear stress and Bradykinin (Fig. 1C). However, in order to reliably study graded shear stress sensing, a higher percentage of cells with a responsive gene network was required. We postulated that the gene network was functional in a low percentage of cells due to inefficient transfection, since transfecting 3 plasmids into a cell at the same time is difficult. We have carried out experiments with sequential transfection, but these failed to deliver positive results. We also systematically modified electroporation parameters (such as pulse voltage, pulse duration, pulse number, cell densities, etc.), but these optimizations failed to increase transfection efficiencies above 20% while maintaining reasonably high cell viabilities. Reagent based transfection methods were also tried, however these delivered lower transfection efficiencies compared to electroporation. Transfection efficiencies were also estimated by electroporating cells with a plasmid set that contained a constitutive GFP expressing plasmid instead of the tTA dependent reporter plasmid (see Fig. S5). However, this method might overestimate transfection efficiencies for the entire gene network, as the constitutive GFP plasmid was capable of producing fluorescence on its own, without the presence of other plasmids and without induction of the gene network (see Fig. S5). For the gene network to work, all gene encoding plasmids needed to be present in the cell, not only the GFP expressing plasmid.

**Figure 2:**
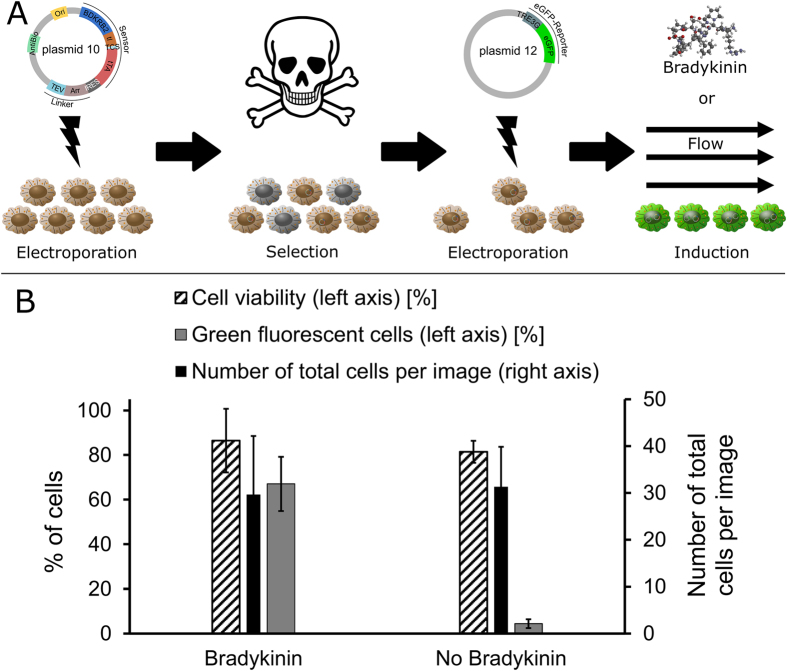
Transfection method for hard-to-transfect mammalian cells. (**A**) Illustration of the developed transfection procedure. Employed mammalian cells are capable of replicating plasmids by expressing the SV40 large T antigen. These cells are first electroporated with the plasmid (p10) which contains the mammalian antibiotic selection (e.g. neomycin) as well as the plasmid replication cassette (SV40 origin of replication). Next, electroporated mammalian cells are selected using 200 μg/ml neomycin (G 418) final concentrations. Selected cells are then electroporated with the second plasmid (p12). Following the second electroporation, cells are induced either by bradykinin or shear stress. (**B**) Green fluorescent cell percentages following the above described transfection procedure. Cells transfected with the method described in part (**A**) were either treated with Bradykinin or left untreated, as shown on the X-axis. Surface-adherent cells were stained with 2 μg/ml Hoechst 33342 and 0.5 μg/ml propidium iodide, fixed with 4% (v/v) paraformaldehyde, microscopy imaged, and counted in ImageJ. Determined green fluorescent cell percentages and cell viabilities are plotted on the left Y-axis alongside total cell numbers per images on the right Y-axis. Error bars represent ± SD, N = 3.

Ultimately, to increase transfection efficiencies, the gene network was redesigned to reduce the number of encoding DNA plasmids from 3 to 2 (Fig. S2B). Furthermore, a mammalian antibiotic selection marker (neomycin), as well as a replication origin (Simian virus 40, SV40) for plasmid multiplication in mammalian cells was also included in one of the two plasmids (Fig. S2B). Simultaneous direct transfection of both plasmids did increase transfection efficiencies up to 30%. Consequently, an enrichment procedure was devised where the plasmid encoding the sensor and linker modules as well as an SV40 plasmid replication and an antibiotic (neomycin) resistance cassette was electroporated into cells, followed by antibiotic (neomycin) selection of plasmid-containing cells that were then further electroporated with the reporter plasmid (Fig. 2A). The SV40 replication system facilitated plasmid replication, thus plasmids with half-lives on the order of hours^37^,^38^,^39^ were made available over the course of several days. Electroporating the improved network into HMEC-1 endothelial cells using the optimized procedure yielded greater than 60% green fluorescent cells when stimulated with Bradykinin (Fig. 2B).

Our integral electroporation, mammalian plasmid replication and mammalian antibiotic selection based transfection procedure takes 1 week, which is short compared to the approximately 6 months required for stable cell line generation^40^,^41^,^42^. This new transfection approach empowered transfection of hard-to-transfect human cells, opening up avenues for pharmacological studies and animal model applications.

### Development of a linear shear stress inducing flow device

**Figure 3:**
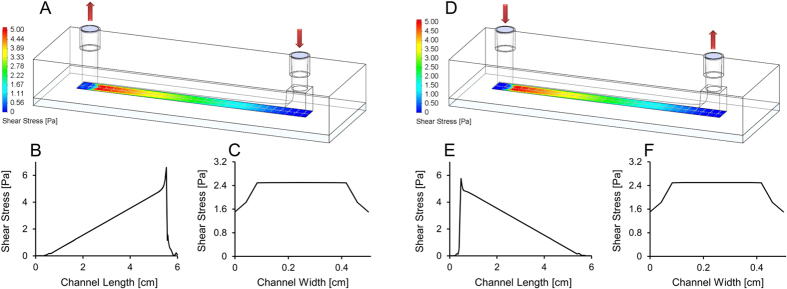
Design and computational validation of the in-house designed linear shear stress inducing flow chamber. (**A**) Geometry of the bespoke height-variance flow channel with shear stress map at the channel floor cell seeding area. Flow entered at the high height end of the channel and exited at the low height end. (**B**) Evolution of shear stress along the length of the channel floor, in the width-wise centre of the channel floor for the channel modelled in part A above. (**C**) Shear stress plotted across the width of the channel floor, in the length-wise centre of the channel floor, for the above modelled channel. (**D**) Geometry of the bespoke height-variance flow channel with reverse flow direction shear stress map at the channel floor. Flow entered at the low height end of the channel and exited at the high height end of the channel. (**E**) Shear stress along the length of the channel floor, in the width-wise centre of the channel floor, for the channel modelled in part D above. (**F**) Shear stress computed across the width of the channel floor, in the length-wise centre of the channel floor, for the channel illustrated in part D above. Flow rate was 10 ml/min in all plots and illustrated models. For shear stress vs. channel length and width plots at 5 ml/min and 20 ml/min see Fig. S6.

In order to effectively apply a graded shear stress response in gene network-encoding cells, a flow chamber with variable height was designed. First, the 3D CAD model of the flow chamber with 0.5 cm width, 5 cm length and height varying along the chamber length described by a square root function, was generated in SolidWorks (Fig. 3A,D). The resultant shear stress on the bottom (cell-seeded) surface of the chamber, computed in SolidWorks’ Flow Simulation Package, increased linearly along the chamber length while shear stress was uniform across the chamber width (Fig. 3B,C,E,F). The chamber was developed and computationally validated for applying wall shear stress in the 0 to 10 Pa range, which can be decreased by lowering the flow rate (Fig. S6). Thus, this flow device facilitates studies in the entire shear stress range of human arteries, including shear stress below 1 Pa in atherosclerosis prone arterial regions and normal veins, between 1 Pa and 7 Pa in normal arteries, and between 7 Pa and 10 Pa in advanced plaques, cardiac valves and stents^43^. To obtain the physical flow channel with this rather complex geometry, two separate parts were used: a glass slide on which cells were seeded and a bottomless flow channel. The bottomless flow channel was obtained by casting polydimethylsiloxane (PDMS) on metal mold parts. Complementary equipment for cell seeding, clamping and microscopy visualization were also designed in SolidWorks and manufactured (Fig. S7). The purpose-built PDMS well was mounted atop of the glass slide to facilitate the seeding of cells on the glass slide. After cell seeding, the PDMS well part could be replaced with the bottomless flow channel to expose cells to shear stress. The complementary clamping and microscopy visualization equipment consisted of two metal plates in between which the bottomless flow channel is mounted on top of the glass slide, and the two metal plates were held together by screws. Both metal plates contained an opening in the channel area to allow light to pass through the equipment for visualization of cells under microscopes. The bottom metal plate was designed to fit into the stage of conventional microscopes.

After the flow channel and complementary equipment was manufactured, the setup was first tested for leakiness and no leakiness was observed. Next, cells were seeded in the PDMS seeding well mounted on the glass slide with the aid of the complementary clamping equipment. Twenty-four hours after cell seeding, the PDMS seeding well was replaced with the bottomless flow chamber and the cell monolayer was exposed to shear stress ranging from 0 to 5 Pa along the chamber length for 24 hours. We validated the functionality of our custom *in vitro* flow device by observing the presence of contamination-free cells after the flow experiment (Fig. S8) and cell alignment to the flow direction at shear stress values above 1.5 Pa (to a maximum of 8 Pa). Using this device, cells can be exposed to an adjustable range of linearly increasing shear stress within a single chamber.

### Monitoring of shear stress sensing

**Figure 4:**
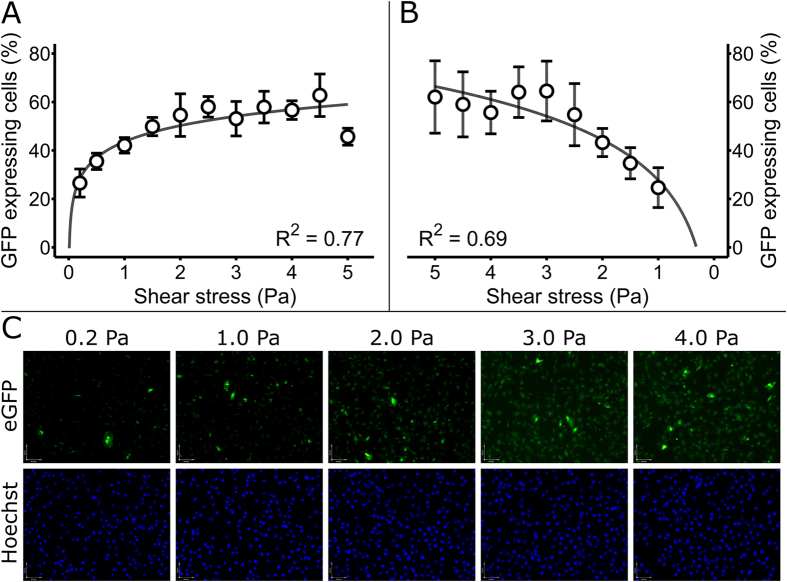
The gene network senses graded levels of shear stress. (**A**) Graded gene network activation by shear stress. HMEC-1 cells were transfected with the procedure described in Fig. 2 and seeded in the flow channel shown in Fig. 3 for activation by linearly increasing shear stress. Following the flow experiment, cells were stained with Hoechst 33342 and propidium iodide, fixed with paraformaldehyde, and microscopy imaged and counted in ImageJ. Plotted GFP expressing HMEC-1 cell percentages versus shear stress shows a logarithmic relationship (N = 7 flow chambers). Error bars represent ± SD. (**B**) Pilot flow experiment with reversed flow direction (high shear to low shear). Cells were treated as described in part A with the exception of the flow direction being connected in the opposite direction in the flow channel (high shear to low shear). GFP expressing HMEC-1 cell percentages vary logarithmically in function of shear stress (N = 1 flow channel, 4 sets of images). (**C**) Representative fluorescent images of HMEC-1 endothelial cells electroporated with the synthetic network and exposed to different levels of shear stress (based on position within the microfluidic device), as described in part A. Green fluorescence (GFP) indicates gene network activity and blue fluorescence (Hoechst) shows the total number of cells.

To explore the sensitivity of our gene network to graded levels of shear stress, HMEC-1 endothelial cells were electroporated with the gene network (encoded on p10 and p12, Table S1), using the above described transfection procedure (Fig. 2) and were seeded in the custom flow chamber for exposure to the shear stress range of 0 to 5 Pa for 24 hours. Cells were then fixed and the entire length of the chamber was imaged using fluorescence microscopy (Fig. 4C). Results showed that increasing shear stress caused accumulative G-PCR activation that was best fit by a logarithmic function (Fig. 4A). As a consequence, the G-PCR displayed a higher sensitivity to discriminate changes in shear stress at physiological values of 0 to 2 Pa. To eliminate the possibility that release of signaling molecules by cells in the upstream (low shear) region of the chamber might affect the activation of the gene network in cells of the downstream (high shear) region, we reversed the flow direction in the device. Results showed a logarithmic relationship between increasing shear stress and G-PCR activation (Fig. 4B), similar to the case of direct flow experiments. Analysis of covariance (ANCOVA) did not show differences between the two logarithmic graphs, proving that the increase in shear stress, and not paracrine signaling, caused the increase in G-PCR activation. To the best of our knowledge, this is the first synthetic network capable of detecting a graded response to shear stress in hard-to-transfect endothelial cells.

The 0 to 5 Pa shear stress range was chosen due to its physiological relevance. Shear stress values between 1.5 and 2 Pa are regarded as physiological or normal in healthy large human arteries^44^,^45^. Shear stress below this physiological range is considered to promote the development of atherosclerosis^43^,^46^,^47^,^48^,^49^, which is the main contributor to cardiovascular diseases and the single leading cause of human mortality worldwide^50^,^51^. A shear stress magnitude greater than 2 Pa can occur under diseased conditions, for example when the diameter of the artery is reduced by the atherosclerotic plaque and flow velocities are consequently high^45^,^52^. The upper limit of the shear stress range of 5 Pa was determined based on flow experiments with HMEC-1 endothelial cells where cells washed away at shear stress magnitudes above this limit. However, our custom flow chamber is capable of exposing cells to shear stress magnitudes of up to 10 Pa (cf. Fig. S6). Therefore, our platform (gene network and flow device) can be applied to a large variety of mechanotransduction studies, including low atherogenic shear stress, physiological and diseased high shear stress.

### Gene expression modulation by mechanical stimuli

**Figure 5:**
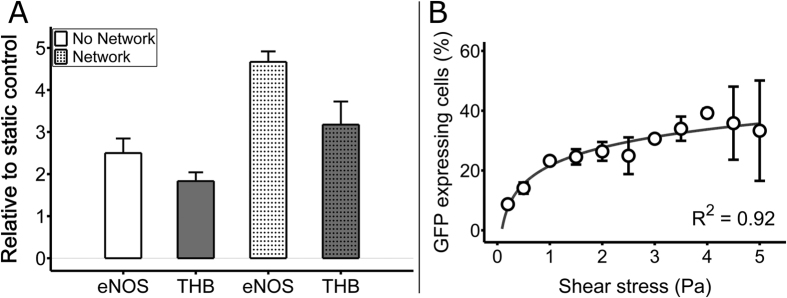
Gene expression modulation by mechanical stimuli. (**A**) The KLF2-eGFP fusion protein of the gene network up-regulates downstream eNOS and THB genes. HMEC-1 cells were either transfected with the gene network following the method describe in Fig. 2, or left untransfected. All cells were exposed to a shear stress of 2 Pa for 24 hours. Next, eNOS and THB mRNA levels were quantified relative to untransfected static (i.e., no flow) controls. Two-way ANOVA showed a statistical increase (p < 0.0001, N = 3) in the mRNA levels of both eNOS and THB in gene network transfected flow exposed cells compared to flow exposed cells without the gene network. (**B**) KLF2-eGFP expression modulation by shear stress in HMEC-1 cells. Green fluorescent cell percentages vary logarithmically as a function of shear stress (N = 3 flow chambers). All error bars represent ± SD.

We subsequently modified the network to modulate the expression of a known mechanosensitive transcription factor. We chose Krueppel like factor 2 (KLF2) because it is an important mechanotransduction transcription factor that regulates greater than 50% of mechanosensitive genes in endothelial cells^53^,^54^,^55^. Towards this end, the existing reporter plasmid (p12) was modified by replacing the eGFP gene with a KLF2-eGFP fusion gene (Fig. S9). The gene network (encoded on p10 and p14) was electroporated into HMEC-1 endothelial cells and functionality of the network was first assessed by exposing the cells to increasing concentrations of Bradykinin, which activated expression of KLF2-eGFP in a dose-dependent manner (Fig. S10). To validate that the KLF2 gene within the synthetic network behaves similarly to endogenously expressed KLF2, particularly with the addition of the fused GFP tag, HMEC-1 cells were either electroporated with the network or left untransfected and the mRNA levels of two genes (endothelial nitric oxide, eNOS, and thrombomodulin, THB) known to be upregulated by KLF2^54^,^56^,^57^ were examined after 24 hours of flow relative to the mRNA levels in untransfected static (i.e., no flow) controls (Fig. 5A). A two-way analysis of variance (ANOVA) was used to assess the influence of the network and gene type on gene expression within the different cell populations. Presence of the gene network caused a statistically significant increase in expression of eNOS and THB compared to untransfected cells (*p* < 0.0001, N = 3). In addition, THB mRNA levels were statistically higher (*p* < 0.001, N = 3) than eNOS mRNA levels. No statistically significant interaction was found between the network and gene type variables (*p* = 0.086). To examine whether we could monitor the activity of KLF2 over increasing levels of shear stress, the cells were seeded into our custom microfluidic device and exposed to the same flow regimen described above. Similar to our original synthetic network, results showed increasing levels of GFP with increasing shear stress that was best described by a logarithmic function, demonstrating that we could directly modulate KLF2 expression by shear stress (Fig. 5B).

## Conclusions

Our experiments demonstrate that a novel synthetic mechanosensitive gene network can monitor the level of shear stress acting on mammalian cells. In addition, we were able to modify the network to directly regulate the atheroprotective transcription factor KLF2, which bypassed the upstream mitogen-activated protein kinase signaling pathway^58^ and directly modulated downstream signaling through the synthetic network. Our approach opens up avenues to rewire cell processing of disease-promoting mechanical stimuli into therapeutic outputs in mouse models, although the details of genetic material insertion into embryonic murine cells fall outside the scope of the study presented here. Since the synthetic network is activated by high shear stress, it could be also used to screen compounds that seek to activate atheroprotective signaling pathways in the presence of low and oscillatory shear stress, which are known atherogenic mechanical stimuli^59^. Additionally, new mechanosensitive G-PCRs could be identified by replacing the current G-PCR with a potentially mechanosensitive one (e.g., a G-PCR that is expressed in mechanosensitive cells). More generally, the modular nature of the network entails that components can be replaced to monitor virtually any gene in the mechanotransduction signaling cascade to screen pharmaceutical targets against pathological mechanical signaling. Finally, the developed flow setup and transfection procedure is useful for researchers from within and outside the field of mechanosyngenetics.

## Materials and Methods

### Plasmid construction

DNA sequences were constructed *in silico* using SnapGene Viewer. *De novo* DNA synthesis of double stranded DNA was outsourced to Eurofins NWG Operon, Ebersberg, Germany. Received plasmids with synthetic DNA were transformed into competent *Escherichia Coli* (*E. coli*) for amplification and storage. *E. coli* cultures of strains JM109, NEB 5α F’Iq and DH5α were grown in Luria-Bertani (LB) broth at 37 °C at 180 RPM. *E. coli* colonies were grown on LB-Agar containing Petri dishes supplemented with appropriate antibiotics. Competent JM109 and DH5α *E. coli* cells were prepared in-house and subsequently transformed using the heat shock method. DNA plasmids were extracted from *E. coli* and purified using the QIAprep Spin Miniprep Kit from Qiagen, following the manufacturer’s protocol. DNA fragments were amplified from DNA plasmids using Polymerase Chain Reaction (PCR). DNA stands were separated using 1% agarose gel electrophoresis. DNA was extracted from the agarose gel using the QIAquick Gel Extraction Kit from Qiagen, following the Qiagen protocol. For salt sensitive applications (e.g. blunt end ligation) linear DNA fragments were purified using the QIAquick PCR Purification Kit from Qiagen. DNA was cut and ligated using conventional restriction enzyme and DNA ligases, respectively. Obtained DNA sequences were verified using Sanger sequencing carried out at GATC Biotech AG, Konstanz, Germany. Primers for sequencing and PCR were designed using SeqBuilder from DNASTAR, Lasergene. For storage plasmid containing *E. coli* cells were frozen in 20% (v/v) glycerol solution in −80 °C freezers. DNA plasmids for transfections into mammalian cells were purified using the EndoFree Plasmid Mega or Giga Kits from Qiagen, following Qiagen’s protocol.

### Mammalian cell line culture

EA.hy926 cells were cultured in Dulbecco’s Modified Eagle’s Medium (DMEM) supplemented with final concentrations of 5 mM L-Glutamine, 10 mM 4-(2-Hydroxyethyl)piperazine-1-ethanesulfonic acid (HEPES), 10% (v/v) foetal bovine serum (FBS) and, optionally, 100 units/ml penicillin with 100 μg/ml streptomycin. HeLa were grown in DMEM supplemented with final concentrations of 5 mM L-Glutamine, 10 mM HEPES, 10% (v/v) foetal bovine serum (FBS) and, optionally, 100 units/ml penicillin with 100 μg/ml streptomycin. The immortalized human microvascular endothelial cell line (HMEC-1) were grown in MCDB 131 medium supplemented with final concentrations of 15% (v/v) FBS, 1 μg/ml hydrocortisone, 10 ng/ml endothelial cell growth factor (ECGF), 2 mM L-glutamine, and, optionally, 100 units/ml penicillin and 100 μg/ml streptomycin. All cell types were cultured at 37 °C, saturated humidity and 5% (v/v) CO_2_ in Corning polystyrene cell culture flasks with 25 cm^2^ or 75 cm^2^ CellBIND cell growth surface area. For storage, mammalian cells were frozen in 10% dimethyl sulfoxide (DMSO) in cryovials in liquid nitrogen. All methods in this study were carried out in accordance with the relevant guidelines and regulations. All the used materials and experimental protocols were approved by Department of Bioengineering, Imperial College London, following the Health and Safety Act of 1974. The immortalized human umbilical vein endothelial cell line (EA.hy926) was kindly provided by Dr. Beata Wojciak-Stothard from the Department of Experimental Medicine and Toxicology, Imperial College London. The immortalized human microvascular endothelial cell line (HMEC-1) was generously supplied by Ann Mccormack and Dr. Adrian H Chester from the National Heart & Lung Institute, Imperial College London. HeLa cells were obtained from the American Type Culture Collection (ATCC).

### Electroporation transfection

For plasmid electroporation into mammalian cells, the Neon™ Transfection System from Life Technologies Inc. with 10 μl electroporation tips was used following the manufacturer’s instructions. Pulse voltage (500–1500 V), pulse duration (5–500 ms), number of pulse (1–20) and cell electroporation density (50,000–300,000 cells per 10 μl) were optimized individually for each cell type.

### Mammalian antibiotic selection

The optimal neomycin (G 418) antibiotic concentration for selection was determined by culturing mammalian cells under antibiotic concentrations increasing by increments of 100 μg/ml, up to 1500 μg/ml. In the optimized mammalian antibiotic selection procedure HMEC-1 cells were electroporated with the p10 plasmid, 24 hours after electroporation 200 μg/ml of G 418 was added, 48 hours after G 418 addition G 418 was removed. Twenty-four hours later, cells were electroporated with the p12 plasmid and 24 hours after the second electroporation the gene network was induced.

### Induction and inhibition of the gene network

To induce the expressed gene network, 6 hours after electroporation, 2 μM of [Hyp^3^]-Bradykinin and 2 μM of Bradykinin^60^,^61^,^62^,^63^ was added to the culture, re-added every 6–12 hours until the following day when the cells were imaged. To induce the gene network by applying well controlled shear stress, flow experiments were carried out using in-house developed flow setups (see below). To inhibit the gene network at the shear stress sensor level, 20–100 nM of the HOE 140 selective B2 Bradykinin receptor antagonist was added 1–4 hours after electroporation^28^,^29^,^30^. To inhibit the gene network at the transcription factor (tTA) level, 20 ng/ml doxycycline was supplied 10 minutes after electroporation^32^,^33^.

### Design and manufacturing of linear shear stress inducing flow chamber

A 3-Dimensional Computer Aided Design (3D CAD) model of a 0.5 cm wide, 5 cm long flow chamber with a height varying along the chamber length described by a square root function, was generated in SolidWorks 2012. The shear stress at the bottom wall of the chamber (chamber floor) in the resulting model was calculated using the Flow Simulation 2012 Computational Fluid Dynamics package in SolidWorks. For validation, obtained flow velocities were compared to analytically calculated flow velocities.

The bottomless flow chamber was manufactured by polydimethylsiloxane (PDMS) casting on metal mold patterns contained inside a petri dish. The curved chamber height profile of the metal mold was generated by high precision wire-cut electric discharge machining (EDM) using a Fanuc Robocut α-oic wire-cut electric discharge machine. The obtained geometry was verified with a Xyris 4000 Confocal Laser Surface Profiler from Taicaan Technologies. Complementary equipment for clamping and microscopy visualization was also manufactured.

### Flow experiments

The flow setup consisted of a peristaltic pump, a bubble catcher media reservoir, platinum-cured silicone tubing and a flow chamber. Cells were seeded at a density of 40000 cells/cm^2^ on a 7.5 × 2.5 cm standard plain microscopy glass slide coated with 1 mg/ml fibronectin solution. The bottomless PDMS chamber was mounted atop of the glass slide and held together with the complementary clamping equipment. The flow experiment was started by priming cells for 1 hour at 0–0.5 Pa shear stress, then the flow rate was gradually increased to yield the experimental 0.2–5 Pa shear over the following 2 hours and then maintained at the experimental level for 24, 36 or 48 hours. During flow experiments the culture media was flowing from the media reservoir, through the flow chamber to the pump and back to the media reservoir.

### Cell staining, microscopy imaging and image processing

After the flow experiments, cells were stained with 1 μg/ml propidium iodide to identify dead cells and with 2 μg/ml Hoechst 33342 to determine the total number of cells. Following staining, cells were fixed with 4% paraformaldehyde (PFA). Cells were visualized under Leica DM IL LED–DFC295 or Leica DM IL LED–DFC290 inverted phase contrast microscopes. Cells were imaged using a Hamamatsu ORCA-ER camera coupled to a Zeiss Axiovert 200 inverted fluorescent microscope with a fully motorised stage, controlled by Improvision Volocity acquisition software. To count cells and determine transfection efficiencies as well as cell viabilities, the acquired microscopy images were processed in ImageJ 1.47^34^,^35^. See SI Text for further details on materials and methods used.

## Additional Information

**How to cite this article**: Kis, Z. *et al*. Development of a synthetic gene network to modulate gene expression by mechanical forces. *Sci. Rep.* **6**, 29643; doi: 10.1038/srep29643 (2016).

## Supplementary Material

Supplementary Information
