# Supplementary material for: Development of a synthetic gene network to modulate gene expression by mechanical forces
Source: Sci Rep. 2016 Jul 12;6:29643. doi: 10.1038/srep29643 (PMC4940741; doi:10.1038/srep29643)
Supplement: Supplementary Information [file srep29643-s1.pdf]

Development of a synthetic gene network to modulate gene expression by mechanical forces

Zoltán Kis, Tania Rodin, Asma Zafar, Zhangxing Lai, Grace Freke, Oliver Fleck, Armando Del Rio Hernandez, Leila Towhidi, Ryan M. Pedrigi, Takayuki Homma, and Rob Krams

Supplementary Information

Materials and Methods

Bacterial growth conditions

Bacterial cultures of JM109, NEB 5α F'Iq and DH5α Escherichia Coli (E. coli) strains were grown in Luria-Bertani (LB) broth (1% weight/volume (w/v) tryptone, 0.5% (w/v) yeast extract, 1% (w/v) sodium chloride) at 37°C at 180 RPM in a Labnet 311DS shaking incubator. Colonies were grown on LB-Agar containing Petri dishes (1% (w/v) tryptone, 0.5% (w/v) yeast extract, 1% (w/v) sodium chloride, 1.5% (w/v) agar) supplemented with appropriate antibiotics. Ampicillin (Amp) was added at 50 µg/ml concentration to both for the LB broth and LB-Agar after autoclaving. High efficiency NEB 5α F'Iq competent E. coli cells (C2992H) were purchased from New England Biolabs Inc. (NEB), Ipswich, MA, USA. JM109 and DH5α competent E. coli cells were readily available in our laboratories. LB broth, (Miller BP1426500), LB-Agar, (Miller BP1425500) and ampicillin in form of sodium salt (BP17605) were procured from Fisher BioReagents, Hampton, NH, USA. Petri dishes (101R20, 101VR20 and 101VBLUE) were acquired from Sterilin Ltd., Cambridge, UK. For media, additives and reagent preparation, Milli-Q water with 18.2 MΩ×cm (at 25°C) resistivity produced using Pur1te Select EMD Millipore water purification systems was used. Liquids were sterilized at 121°C for 30 minutes and solids or instruments at 135°C for 5 minutes in a MLS-3751L Sanyo autoclave.

Preparation of competent E. coli cells

A 2 ml pre-culture of *E. coli* strain JM109 or DH5α was prepared by incubation overnight at 37°C at 180 RPM shaker speed. Next, 100 ml LB broth was inoculated with 1 ml of the pre-culture and incubated at 37°C at 180 RPM. When the optical density at 600 nm wavelength (OD_{600}) reached 0.3 absorbance units (AU), the bacterial culture was divided into two 50 ml polypropylene BD Falcon centrifuge tubes with screw cap and placed on ice for 5 minutes. Afterwards the culture was centrifuged at 5,000 ×g for 10 minutes at 4°C. The pellet was re-suspended in 20 ml TfbI per tube using a Fisher Scientific FB15012 vortex mixer or an IKA Lab Dancer S2 shaker, placed on ice for 5 minutes and centrifuged for 10 minutes at 5,000 ×g at 4°C. Pelleted cells were re-suspended in 2 ml of TfbII per tube using a Fisher Scientific FB15012 vortex mixer or an IKA Lab Dancer S2 shaker and placed on ice for 15 minutes. Following this, 100 µl of competent *E. coli* cells were quickly dispensed into separate autoclave-sterilized ice-cold 1.5 ml tubes, frozen quickly in liquid nitrogen and stored at -80°C in a New Brunswick Scientific ultra-low temperature U 725-86 lab freezer for subsequent transformations. TfbI was composed of Milli-Q water with final concentrations of 30 mM potassium acetate, 100 mM RbCl, 10 mM $CaCl_2{\cdot}2H_2O$, 50 mM $MgCl_2{\cdot}4H_2O$ and 15% (volume/volume, (v/v)) glycerol with pH adjusted to 5.8 using 0.2 M acetic acid and sterile filtered. TfbII consisted of final concentrations of 10 mM 3-(N-morpholino)propanesulfonic

acid (MOPS), 75 mM $CaCl_2 \cdot 2H_2O$, 10 mM RbCl and 15% (v/v) glycerol in Milli-Q water, pH adjusted to 6.5 with KOH and sterile filtered. Potassium acetate (60035), RbCl (R2252-50G), $CaCl_2 \cdot 2H_2O$ (C3306-100G), MOPS (69947-100G), acetic acid (A6283), KOH (38073) and $MgCl_2 \cdot 4H_2O$ (221279) were purchased from Sigma-Aldrich Corp., St. Louis, MO, US. Glycerol (G065017) was obtained from Thermo Fisher Scientific Inc. 50 ml polypropylene BD Falcon conical, high-clarity centrifuge tubes with blue polyethylene flat-top screw caps (352070) were purchased from Thermo Fisher Scientific Inc. For filtration cellulose acetate syringe filters (28145-477) with 0.2 µm pore size have been used, from Sartorius Stedim Biotech, Aubagne, France. Ice was generated using the Scotsman AF20ASE Ice flaker. Milli-Q water with 18.2 MΩ×cm (at 25°C) resistivity was produced by Pur1te Select EMD Millipore water purification systems.

In silico **design of plasmid constructs and** ***De novo*** **DNA synthesis**

DNA sequences of the desired gene constructs were constructed *in silico* using SnapGene Viewer and Microsoft Word 2010 using sequences from the National Center for Biotechnology Information (NCBI) GenBank and sequences provided by Dr Barnea. These sequences served as a template for DNA sequencing primer design and for DNA synthesis. *De novo* DNA synthesis of double stranded DNA was carried out at Eurofins NWG Operon, Ebersberg, Germany and synthesized genes were delivered in DNA plasmids. Obtained plasmids were transformed into competent *E. coli* for amplification and storage.

E. coli **transformations**

Competent JM109 and DH5α *E. coli* cells were transformed using the heat shock method. For this, 0.1 µl of 1 µg/ml of medium or high copy number plasmid solution was added to 100 µl of competent *E. coli* cell solution and placed on ice for 30 minutes. Afterwards, cells were heat shocked at 42°C for 1 minute and returned to ice for 2 minutes. 1 ml of LB broth containing 0.4% (w/v) D-glucose and 20 mM $MgCl_2$ was added to cells and followed by incubation at 37°C, 180 RPM for 1 hour. Next, 100 µL of the transformed cell solution was plated on Petri dishes containing LB-Agar supplemented with the appropriate selection antibiotic and 0.4% (w/v) D-glucose, and Petri dishes were incubated at 37°C overnight. The remaining transformed cell solution was centrifuged at 13,000 ×g for 10 minutes at 4°C, the pellet was re-suspended in 100 µL LB medium by pipetting and cells were plated on Petri dishes with LB-Agar supplemented with 50 µg/ml Amp and 0.4% (w/v) D-glucose, and Petri dishes were incubated at 37°C overnight. The following day successful transformants were isolated. Similarly, high efficiency NEB 5α F'I^q competent *E. coli* cells were also transformed using the heat shock method by adding 0.1 µl of 0.5 µg/ml of medium or high copy number plasmid solution to 50 µl of competent *E. coli* cell solution and placed on ice for 30 minutes. Next, cells were heat shocked at 42°C for 30 seconds and returned to ice for 5 minutes, without mixing. For recovery, 950 µL of room temperature Super Optimal broth with Catabolite repression (SOC) outgrowth medium was added to cells and cells were incubated at 37°C, 180 RPM for 1 hour. Next, 100 µL of the transformed cell solution was plated on LB-Agar Petri dishes supplemented with 50 µg/ml Amp and 0.4% (w/v) D-glucose, and Petri dishes were incubated at 37°C overnight. The remaining transformed cell solution was centrifuged at 13,000 ×g for 10 minutes at 4°C, the pellet was re-suspended in 100 µL LB medium by pipetting and cells were plated on LB-Agar Petri dishes supplemented with 50 µg/ml Amp and 0.4% (w/v) D-glucose, and Petri dishes were incubated at 37°C overnight. D-glucose (G5500) and $MgCl_2 \cdot 4H_2O$ (221279) were purchased from Sigma-Aldrich Corp. SOC outgrowth medium (B9020S) was acquired from NEB.

Plasmid preparation

DNA plasmids were extracted and purified using the QIAprep Spin Miniprep Kit from Qiagen N.V., Venlo, Netherlands, following the manufacturer's protocol. For this, *E. coli* from single colony from a freshly streaked selective plate was picked and inoculated in 5 ml LB culture containing the appropriate selective antibiotic, where applicable, in 14 ml polypropylene BD Falcon tubes with snap cap. The liquid LB culture was incubated overnight (12–16 hours) at 37°C with shaking at 180 RPM. Bacterial cells were harvested by centrifugation at 4,000 x g for 10 min at 4 °C in a Hettich Rotanta 460R large bench centrifuge. The cell pellet was re-suspended using a Fisher Scientific FB15012 vortex mixer or an IKA Lab Dancer S2 shaker and lysed under alkaline conditions [1]. The lysate was subsequently neutralized and adjusted to high-salt binding conditions. The solution was applied and plasmid DNA was bound to a QIAprep spin column; the column was washed and then eluted in 50 µL of Milli-Q water to obtain the purified plasmid DNA. Plasmid DNA concentrations and quality were measured using a NanoDrop 2000c Spectrophotometer, Thermo Fisher Scientific Inc., Waltham, MA, USA. The NanoDrop 2000c Spectrophotometer was blanked with 2 µl of Milli-Q water. 1.5 or 2 µl of plasmid DNA sample was loaded on the NanoDrop 2000c instrument and DNA concentrations were always measured 3 times and the average concentration was used. To evaluate the quality of plasmid DNA solution using the NanoDrop 2000c Spectrophotometer [2], the ratio of absorbance at 260 nm and 280 nm was confirmed to be above 1.8, indicating pure DNA (without protein, phenol or other contaminants that absorb strongly at or near 280 nm). Similarly, the absorbance ratio at 260 nm and 230 nm was also checked to be in the 2.0 – 2.2 range, characteristic to pure DNA (free on contaminants that absorb at 230 nm). Milli-Q water with 18.2 MΩ×cm (at 25°C) resistivity was produced using Pur1te Select EMD Millipore water purification systems. 14 ml polypropylene BD Falcon tubes (352059) with round bottom and dual-position snap cap were purchased from Thermo Fisher Scientific Inc.

Primer design and DNA sequencing

Plasmids (listed in **Table S1**) were transformed into competent *E. coli* cells, prepared (as described above) and Sanger sequenced at GATC Biotech AG, Konstanz, Germany on Sanger ABI 3730xl instruments. GATC's DNA sequencing technique from is ISO 9001 certified. Sanger sequencing is considered the "gold standard" with the longest read lengths, of up to 1,100 bases. The Sanger ABI 3730xl is a 96 capillary instrument with 50 cm capillaries for providing high throughput analysis and high resolution. Robust sequencing chemistry was assured by BigDye Terminator v3.1 and for GC-rich samples by dGTP BigDye Terminator Cycle Sequencing Kit. GATC's laboratory information management system offered fast and secure data transfer. For DNA sequencing, primers were designed using SeqBuilder Version 9.1.0 (109) from DNASTAR, Lasergene using sequences from National Center for Biotechnology Information (NCBI) GenBank as a template. The primers were generally designed 21 nucleotide long, with GC content of about 40-60%, with melting temperatures between 52-58 °C (this range generally yields best results), with GC clamp at the 3' end (for stronger binding at the 3' synthesis end, though triple H-bonds between bases), avoiding mono- and di-nucleotide repeats and secondary structure artifacts such as hairpins, and self- and cross- dimerization products. Synthesized primers received in powder state, were briefly centrifuged to collect the powder at the bottom of the tube, reconstituted to 100 µM stock solutions, mixed by vortexing to completely dissolve the powder and again briefly centrifuged to collect the solution at the bottom of the tube before freezing at -20°C. Twenty µl of 10 µM solutions were sent to GATC Biotech AG for up to 8 sequencing reactions. Volumes of 20 µL of plasmid DNA with concentration of 80 ng/µL were sent for DNA sequencing for up to 8 sequencing reactions. The obtained DNA sequences were analysed using SeqMan Pro Version 9.1.0 (109), 418 from DNASTAR, Lasergene by aligning with coding sequences from the online GenBank NCBI database. DNA sequences obtained through DNA sequencing were also aligned with sequences from the online GenBank NCBI database using SerialCloner 2 and the T-Coffee online alignment tool

(available at http://tcoffee.crg.cat/apps/tcoffee/do:regular). To exclude codon usage biases in case of point mutations obtained from DNA sequencing, DNA sequences were converted into amino acid sequences using online converters from http://web.expasy.org/translate/, http://bioinformatics.picr.man.ac.uk/research/software/tools/sequenceconverter.html and http://www.fr33.net/translator.php and amino acid sequences from sequencing were compared with amino acid sequences from GenBank NCBI database. Antiparallel, complement and inverse DNA sequences were obtained using the sequence editor available at http://www.fr33.net/seqedit.php. Desired DNA sequences of our gene constructs were generated *in silico* by inserting or replacing the appropriate DNA sequences to or in the corresponding vectors, respectively.

Bioinformatics analysis of GPCR sequences and structures

In order to estimate whether shear stress responsive GPCRs presents obvious N and C – termini shear stress sensing motifs, a series of bioinformatics tools were employed. GPCR amino acid sequences were obtained from UniProt [3], NCBI Protein [4] and RCSB PDB [5] databases. The GPCR sequences were aligned using seeded guide trees and hidden Markov model (HMM) [6] profile-profile techniques as implemented in the Clustal Omega multiple alignment online software [7,8]. The following consensus symbols and colors were used to mark residues that are fully conserved, present strongly-similarity or weakly-similar properties: "* red", ": blue" and ". green", respectively. Protein topologies were modeled with the hidden Markov model applied for transmembrane proteins (TMHMM) [9,10] and with Phobius online software [11,12]. The tertiary structure of the 6 shear stress sensitive GPCRs was predicted with protein homology based algorithms as implemented in Phyre [13]. Resulting GPCR tertiary structured were aligned in PyMOL [14] and sequence similarities were noted using symbols and color codes described above.

Freezing *E. Coli* cells

Cells to be frozen were grown in 3 ml over-night culture. Approximately 1.4 ml of overnight culture was added to cryovials that contained 0.5 ml 80% (v/v) glycerol. Cryovials were stored in the -80°C New Brunswick Scientific ultra-low temperature U 725-86 lab freezer. Fisherbrand polypropylene 2 ml cryovials (12567501) were acquired from Thermo Fisher Scientific Inc.

Polymerase Chain Reaction

For molecular cloning using conventional methods and Gibson Assembly (described below), DNA fragments were amplified from DNA plasmids using Polymerase Chain Reaction (PCR). For PCR, *Taq,* Phusion High-Fidelity (HF), Q5 High-Fidelity, Q5 Hot Start High-Fidelity DNA polymerases and the KAPA 2G Robust HotStart Ready Mix PCR Kit were used. The composition of the PCR mixtures was in accordance with PCR kit manufacturer's protocols. PCR was thermal cycled in Labnet MultiGene™ Gradient PCR Thermal Cycler (TC9600-G-230V) or in Eppendorf realplex^4 epgradient S qPCR Mastercycler, using programs recommended by the PCR kit providers. For the amplification of long DNA fragments (> 6 kb), extension times were increased to 40–50 seconds/kb. DNA primers for PCR were designed using SeqBuilder Version 9.1.0 (109) from DNASTAR, Lasergene or using NEBuilder (available at: http://nebuilder.neb.com/) or using In-Fusion primer design tool (available at: http://bioinfo.clontech.com/infusion/convertPcrPrimersInit.do). Primer synthesis was ordered from Sigma-Aldrich Corp. and from Life Technologies Inc., Carlsbad, CA, USA. Annealing temperatures (ATs) were calculated using NEB Tm Calculator [15] which used nearest-neighbour thermodynamic parameter set from Santa Lucia [16] applying the salt correction algorithm outlined in Owczarzy et al. [17]. For Phusion DNA polymerases, the thermodynamic data were from Breslauer et al. [18]. If the PCR did not yield positive results, a

gradient of ATs ranging ±10 °C (upper limit being 72 °C) of the estimated AT, in increments of 2 °C was used, and the enzyme system was replaced. If PCR was still unsuccessful, the above mentioned gradient PCR was repeated with increased DNA polymerase concentrations in increments of 20% of the initial concentration, to up 1 order of magnitude above the initial concentration. *Taq* (M0273S), Phusion High-Fidelity (M0530S), Q5 High-Fidelity (M0491S) and Q5 Hot Start High-Fidelity (M0493S) DNA polymerases were acquired from NEB. DNA polymerases were supplied with their corresponding reaction buffers, dimethyl sulfoxide (DMSO) and Q5 High GC Enhancer solutions. Deoxyribonucleotide triphosphate (dNTP) Mix, 10 mM each, (R0191) was purchased from Thermo Fisher Scientific Inc. The KAPA2G Robust Hot Start Ready Mix PCR Kit (KK5702) was obtained from Kapa Biosystems, Inc. MA, USA. As nuclease free water both Milli-Q water generated by Pur1te Select EMD Millipore water purification systems and Nuclease-Free Water (129114) from Qiagen N.V. was used.

Agarose Gel Electrophoresis

DNA length and quality was assessed by 1% agarose gel electrophoresis (AGEP) using the Owl™ EasyCast™ B1 Mini Gel Electrophoresis System or the Owl™ EasyCast™ B2 Mini Gel Electrophoresis System from Thermo Fisher Scientific Inc. The 1% agarose solution was prepared by mixing 1 g or 1.5 g of agarose powder in 100 ml or 150 ml 0.5× Tris-Borate-EDTA (TBE) solution, respectively. This mixture was then heated in the microwave oven for approximately 4 min until the agarose was completely dissolved, resulting in a clear solution. The agarose solution was then kept at 55°C in a Grant Instruments GD120 stirred water bath for 20 min. Next, 1 µl of SYBR Safe DNA Gel Stain was added per 10 ml agarose solution. The solution was mixed and poured on a gel tray placed in the casting position in the buffer chamber with combs in place and incubated at room temperature for 1 hour until the gel solidified. Alternately, for gel solidification, the gasket ends of the tray were also fixed and sealed with 4-5 layers of autoclave tape and the liquid gel was poured into this sealed tray without using the buffer chamber. The solidified gel was placed in the electrophoresis chamber in the Owl™ EasyCast™ Mini Gel Electrophoresis System and was poured onto with 0.5×TBE solution to the level indicated on the electrophoresis system. Ten µl of the 6x Orange DNA dye was added per 50 µl of DNA sample solution. Next, 20-60 µl of DNA sample – DNA loading dye mix was loaded into the wells of the gels, depending on the comb size used. Five µl and ten µl of the 1 kbp DNA ladder were also loaded in wells adjacent on both sides to wells containing the DNA sample – DNA loading dye mix. Next, the Owl™ EasyCast™ Mini Gel Electrophoresis Systems containing the gel was connected to the output jacks of a VWR 250 or Fisherbrand Power 300 power supplies. The DNA was ran in the gel from the negative towards the positive electrode for about 40-120 minutes at constant voltage in the range of 120-200 V. Lower voltages were used in the case of Owl™ EasyCast™ B1 Mini Gel Electrophoresis System and in case of preparative AGEP when the DNA was subsequently extracted and used for subsequent enzymatic reactions (e.g. restriction digestion and ligation). For preparative AGEP TopVision low melting point agarose was used. The electrophoresis was stopped when the Orange G of the 6x Orange DNA dye reached the end of the gel, knowing that the speed at which Orange G migrates through the agarose gel is approximately equivalent to 50 bp DNA fragments. After stopping electrophoresis, the DNA containing gel was visualized under low intensity 254nm UV light using the UVP BioSpectrum 500 Imaging System. DNase and RNase free Agarose (BP1356-100) and TopVision Low Melting Point Agarose (R0801) was purchased from Thermo Fisher Scientific Inc., DNase and RNase free agarose (A9539-250G) was also acquired from Sigma-Aldrich Corp. Low electroendosmotic agarose (A0169-10G) and Tris-Borate-EDTA buffer powder blend (T3913) was purchased from Sigma-Aldrich Corp. SYBR safe DNA Gel Stain (S33102) was obtained from Life Technologies Inc., 1 kbp DNA ladder (N3232S) was provided by NEB and 6x Orange DNA loading Dye (RO631) was ordered from Thermo Fisher Scientific Inc.

Extraction of DNA fragments from agarose gel

After imaging the gel, gel bands containing DNA with the size of interest were cut from the using 0.13–0.17 mm thick microscopy cover slips. DNA from the excised bands was subsequently purified using the QIAquick Gel Extraction Kit (28706) from Qiagen N.V. together with an Eppendorf 5415R or a VWR Galaxy 1814 microcentrifuge, following Qiagen's manual. For this, the gel was weighed on a MH-124 analytical balance from Fisher Scientific. Next, the gel was dissolved in 3 gel volumes of solubilisation Buffer QG, assuming a gel density of 1 g/ml, and the gel was heated at 50°C for 10 minutes using GRANT QBD1 or Techne Dri-block DB-2D heating blocks, vortexing every 3–5 minutes to enhance solubilisation. After solubilisation, the DNA solution was loaded onto the silica membrane containing spin column; high chaotropic salt concentrations [19] and a pH ≥7.5 of Buffer QG ensured that the DNA remained bound to the column while contaminants passed through. Next, impurities were washed away by the ethanol-containing Buffer PE, then the ethanol was removed by an additional centrifugation step and the DNA was eluted in nuclease free water. Following elution, DNA concentrations were measured and DNA quality was assessed using a NanoDrop 2000c Spectrophotometer. As nuclease free water both Milli-Q water generated by Pur1te Select EMD Millipore water purification systems and Nuclease-Free Water (129114) from Qiagen N.V. was used. Microscopy cover slips (MNJ-350-110G) were purchased from Gerhard Menzel GmbH. Brunswick, Germany.

Purification of DNA from PCR or from other enzymatic reactions

For salt sensitive applications (e.g. blunt end ligation) linear DNA fragments obtained from PCR or restriction enzyme digestions were purified using the QIAquick PCR Purification Kit (28706) from Qiagen N.V. together with an Eppendorf 5415R or a VWR Galaxy 1814 microcentrifuge, following Qiagen's protocol. For this, the DNA solution was mixed with 5 DNA solution volumes of Buffer PB which contains high chaotropic salt concentrations [19] and a pH ≥7.5, necessary for adsorption of DNA to the silica membrane of the spin columns. After DNA binding, impurities from the column were washed away by ethanol-containing Buffer PE, and then the ethanol was removed by an additional centrifugation step. Next, the DNA was eluted in nuclease free water and DNA concentrations and quality were assessed using a NanoDrop 2000c Spectrophotometer. Nuclease-Free Water (129114) was obtained from Qiagen N.V. or generated using Pur1te Select EMD Millipore water purification systems.

Plasmid construction using conventional cloning

Plasmids or PCR products (with restriction enzyme sites inserted by extensions in PCR primers) were digested using restriction NEB enzymes, following enzyme specific NEB protocols, using supplied buffers (NEBuffer 1, NEBuffer 2, NEBuffer 3, NEBuffer 4) and bovine serum albumin, as required. CutSmart™ Buffer (B7204S) was also purchased from NEB. Resulting linear DNA fragments were separated using Agarose Gel Electrophoresis (AGEP), as described above. DNA was purified from the gel using QIAquick Gel Extraction Kit, as described above. Linear DNA fragments with cohesive (sticky) ends were ligated overnight at 16°C or at room temperature for 10 minutes using the T4 DNA ligase (M0202S) from NEB in 20 µL reaction volumes, following the manufacture's protocol. For linear DNA fragments with blunt ends the room temperature ligation reaction was increased to 2 hours as recommended the by the T4 DNA ligase providing company. When ligating blunt ended DNA fragments, after digestion, the vector fragments were also treated with Thermosensitive Alkaline Phosphatase (M9910) from Promega Corp., WI, USA, by incubating for 15 minutes at 37°C to remove the 5' phosphate groups. The Quick Ligation™ Kit (M2200S) from NEB was also used to ligate linear DNA fragments. After ligation, 4-10 µl of the solution was transformed into competent *E. coli* cells as described above.

Gibson Assembly plasmid construction

To join multiple linearized DNA fragments, the Gibson Assembly was used following the manufactures' instructions. For this, PCR primers with overhangs which were homologous to vector ends were designed and synthesized to amplify insert DNA fragments to be assembled. Using these primers, linear overlapping insert DNA fragments were amplified by PCR and linear vector DNA fragments were obtained by restriction enzyme digestion of circular plasmid vectors. Insert DNA fragments obtained by PCR were purified using the QIAquick PCR Purification Kit Protocol, the concentration was measured using a NanoDrop 2000c Spectrophotometer and aliquots of this solution were ran on AGEP to check the size of the DNA fragments. DNA fragments obtained by restriction digestion were ran on AGEP to separate the vector fragments. Fragments were excised and then extracted from the gel using the QIAquick Gel Extraction Kit, then purified using the QIAquick PCR Purification Kit. The concentration was determined using a NanoDrop 2000c Spectrophotometer. Once the desired overlapping linear DNA fragments were obtained, these were mixed following the protocol from the kit manufacturer.

This reaction mix was incubated in a Labnet MultiGene™ Gradient PCR Thermal Cycler (TC9600-G-230V) or in an Eppendorf realplex^4 epgradient S qPCR Mastercycler at 50°C for 60 minutes. During incubation, the 5' exonuclease enzyme, chewed back 5' ends of the double stranded DNA, creating single-stranded 3´ overhangs that facilitated the annealing of fragments that share complementarity due to extensions added to primers (homologous overlap region). Following annealing, the DNA polymerase filled in gaps and the DNA ligase sealed nicks in the assembled DNA. After the assembly incubation, samples were store on ice or at –20°C for subsequent transformation in competent *E. coli* cells. Transformation was carried out and obtained colonies were brought in liquid LB cultures, plasmids were isolated using the QIAprep Spin Miniprep Kit and were sent for DNA sequencing. Colonies that yielded the desired plasmids were again grown in liquid LB cultures and were stored in 20% glycerol in the -80 °C freezer New Brunswick Scientific ultra-low temperature U 725-86 lab freezer. Gibson Assembly Master Mix (E2611S) was ordered from NEB. The pIRES Vector (631605) was purchased from Clontech Laboratories, Inc.

Production of endotoxin free DNA plasmids for mammalian cell culture use

In order to purify large quantities of plasmids for transfections into mammalian cells, the EndoFree Plasmid Mega (12381) and Giga (12391) Kit from Qiagen N.V. were used following the manufacturer's instruction manual. For this, 2-5 ml of LB medium (containing the corresponding selection antibiotic) was inoculated with the *E. coli* (that contained the plasmid to be purified) from freshly streaked selective plate or from frozen stocks. These pre-cultures were grown over-nigh in 14 ml polypropylene BD Falcon tubes with snap cap at 37°C at 180 RPM in a Labnet 311DS shaking incubator. These starter-cultures were then diluted 1/500 to 1/1,000 to inoculate 500 ml of LB culture (containing the corresponding selection antibiotic) when high-copy number plasmid was purified using the EndoFree Mega kit or to inoculate 2.5 litres of LB culture (containing the corresponding selection antibiotic) when low copy number plasmid was purified using the EndoFree Mega kit. To purify high-copy number plasmid with the EndoFree Giga kit, 2.5 litres of LB culture (containing the corresponding selection antibiotic) were inoculated with 1/500 to 1/1,000 dilutions of the pre-culture. These main cultures were incubated overnight in an IOC400 Gallenkamp orbital shaker incubator in 2 l Erlenmeyer conical polycarbonate flasks (10410482) from Thermo Fisher Scientific Inc. Next day, bacterial cells were harvested by centrifugation in 50 ml polypropylene BD Falcon tubes with screw cap at 4,000 x g for 15 min at 4 °C in a Hettich Rotanta 460R large bench centrifuge. The pellet was then re-suspended using a Fisher Scientific FB15012 vortex mixer or an IKA Lab Dancer S2 shaker and lysed under alkaline conditions. The lysate was cleared by filtration using QIAfilter Mega-Giga Cartridges connected to a Cole Parmer Instruments 35031-10 Aspirator Pump. Next, the endotoxin

removal buffer was added to the clear lysate which was then applied to the QIAGEN Anion-Exchange column for binding under appropriate low-salt and pH conditions. RNA, proteins, dyes, and low-molecular weight impurities were removed by a medium-salt wash. Plasmid DNA was eluted in a high salt buffer. Next the DNA was concentrated and desalted by adding 0.7 volume of room-temperature (to prevent salt precipitation) isopropanol at centrifuged at 15,000 g for 30 minutes at 4°C (to prevent overheating of the sample). Alternatively, this isopropanol precipitation was also carried out by centrifugation at 4,500 g for 60 minutes at 4°C, without modifying the other parameters. The resulting DNA pellet was then washed with endotoxin free room-temperature 70% ethanol and centrifuged at 15,000 g for 10 minutes or at 4,500 g for 60 minutes at 4°C. The pellet was air-dried for 20 minutes under a Bioquell Class II safety cabinet and resuspended in endotoxin-free Buffer TE or endotoxin-free water.

Mammalian cell culture

The immortalized human umbilical vein endothelial cell line (EA.hy926) was kindly provided by Dr. Beata Wojciak-Stothard from the Department of Experimental Medicine and Toxicology, Imperial College London. EA.hy926 cells were cultured in Dulbecco's Modified Eagle's Medium (DMEM) supplemented with final concentrations of 5 mM L-Glutamine, 10 mM 4-(2-Hydroxyethyl)piperazine-1-ethanesulfonic acid (HEPES), 10% (v/v) foetal bovine serum (FBS) and, optionally, 100 units/ml penicillin with 100 µg/ml streptomycin.

HeLa cells were provided by Dr. Jennifer Frueh, former PhD student in our group, and were originally purchased from ATCC. HeLa cells were grown in DMEM supplemented with final concentrations of 5 mM L-Glutamine, 10 mM HEPES, 10% (v/v) foetal bovine serum (FBS) and, optionally, 100 units/ml penicillin with 100 µg/ml streptomycin.

The immortalized human microvascular endothelial cell line (HMEC-1) was gifted by Ann Mccormack and Dr. Adrian H Chester from the National Heart & Lung Institute, Imperial College London. HMEC-1 cells were grown in MCDB 131 medium supplemented with final concentrations of 15% (v/v) FBS, 1 µg/ml hydrocortisone, 10 ng/ml endothelial cell growth factor (ECGF), 2 mM L-glutamine, and, optionally, 100 units/ml penicillin and 100 µg/ml streptomycin.

The immortalized mouse pancreatic endothelial cell line (MS1, Cat. No. CRL-2279) was purchased from LGC Standards. The MS1 cell line was cultured in DMEM supplemented with 5 mM L-Glutamine, 10 mM HEPES, 20% (v/v) FBS and, optionally, 100 units/ml penicillin with 100 µg/ml streptomycin.

All cell types were cultured in MCO-18M, MCO-18AC or MCO-18AIC Sanyo incubators at 37°C, saturated humidity and 5% (v/v) CO_2 in Corning polystyrene cell culture flasks with 25 cm^2 or 75 cm^2 CellBIND cell growth surface area. Cells were passaged and handled in Euroclone Bioair Top Safe 1.5 and Euroclone Bioair Top Safe 1.2 Class II safety cabinets. Prior passaging, the culture media, 1 x PBS and trypsin solution was pre-warmed to 37°C in a Thermo Fisher Scientific DMU19 Water Bath. For passaging, all cell types were washed twice with 1 ml of 1 x PBS solution per 10 cm^2 of cell growth area and were detached by incubating at 37°C for 3-6 minutes in 0.4 ml of 0.25% (w/v) trypsin, 0.02% EDTA solution per 10 cm^2 of cell growth area. Next, 10 ml of culture medium with serum was added per 1 ml of cell-trypsin solution to neutralize the trypsin, and this solution was transferred to 15 or 50 ml conical polypropylene CentriStar™ capped tubes to centrifuge for 5 minutes at 220 g in a MSE Mistral 2000 centrifuge. The resulting pellet was re-suspended in fresh culture medium, transferred to new cell culture flasks and placed back in the cell culture incubator. Liquid volumes in the order of millilitres were handled using 5 ml (734-1737), 10 ml (734-1738) and 25 ml (734-1739) polystyrene Stripettes attached to Integra Pipetboys. For microliter volumes, 10 µl (S1120-3810), 200 µl (S1120-8810), 1,000 µl (S1126-7810) StartLab TipOne Filter Tips were used connected onto Eppendorf Research plus, adjustable-volume, single

channel pipettes. For disposal, liquids were aspirated through autoclaved glass Pasteur pipettes (612-1701, VWR) and C-Flex tubing (190-947-001, Cole-Parmer) into a Büchner flask to be neutralized by Klorsept 87 solution (CLE1490, Scientific Laboratory Supplies Ltd.). The Büchner flask was connected through Whatman Vacu-Guard Filters (09-744-75, Thermo Fisher Scientific Inc.) to a vacuum line powered by a central vacuum pump. Surfaces were sterilized using 70% (v/v) ethanol solution in Milli-Q water with 18.2 MΩ×cm (at 25°C) resistivity. Ethanol absolute AnalaR NORMAPUR (20821.321) was ordered from VWR BDH Prolabo. Conical 15 ml (734-1867) or conical-skirted 50 ml (734-1876) polypropylene CentriStar™ tubes with high-density polyethylene caps were ordered from VWR. Rectangular Corning polystyrene cell culture flasks with 25 cm^2 (734-1712) and 75 cm^2 (734-1713 T75) CellBIND cell growth area, with canted neck and with vented cap were purchased from VWR. L-Glutamine free DMEM (D5546), FBS (F7524), Hydrocortisone (H0888-1G), ECGF (E2759), L-Glutamine (G8540) and Penicillin-Streptomycin solution (P4333), PBS (D8537) and Trypsin-EDTA solution (T4049) were acquired from Sigma-Aldrich Corp. MCDB 131 Medium (10372-019) was purchased from Life Technologies Inc.

Primary cell isolation and culturing

Thoracic aorta segments of 10-15 cm obtained from approximately 6 month old domestic pigs (Sus scrofa domesticus) were obtained within 24 hours of the death of the animal. The aorta segments were stored in Hank’s Balanced Salt Solution (HBSS) supplemented with 200 U/ml Penicillin, 200 µg/ml Streptomycin, 100 µg/ml Gentamycin and 100 µg/ml Amphotericin. Aorta segments and cells were handled in Euroclone Bioair Top Safe 1.5 and Euroclone Bioair Top Safe 1.2 Class II safety cabinets. Intercostal arteries of aorta segments were ligated and the lumen was subsequently rinsed with phosphate buffered saline (PBS) supplemented with 100 U/ml Penicillin, 100 µg/ml Streptomycin, 50 µg/ml Gentamycin and 50 µg/ml Amphotericin to remove red blood cells. Next, one end of the vessel was clamped and the lumen was filled with serum-free Dulbecco's Modified Eagle's Medium (DMEM) containing 0.2 mg/ml collagenase, 200 U/ml Penicillin, 200 µg/ml Streptomycin, 100 µg/ml Gentamycin and 100 µg/ml Amphotericin. The other vessel end was also clamped and the collagenase filled vessel was then incubated at 37°C for 15 minutes. The detached endothelial cells (EC) were collected and the lumen was flushed with PBS supplemented with 100 U/ml Penicillin, 100 µg/ml Streptomycin, 50 µg/ml Gentamycin and 50 µg/ml Amphotericin. The collected ECs were pelleted by centrifuging at 200 g for 5 minutes, re-suspended and placed in culture flasks. The culture medium consisted of DMEM supplemented with 5 mM L-Glutamine, 5 µg/ml EC growth factors, 90 µg/ml heparin and 10% (v/v) foetal calf serum (FCS). Primary pig aortic ECs (PAECs) were cultured for up to 5 passages at 37°C, saturated humidity and 5% (v/v) CO2 in MCO-18M, MCO-18AC or MCO-18AIC Sanyo incubators. Cells were grown in polystyrene Corning cell culture flasks with 25 cm2 (430639) or 75 cm2 (430641) cell growth area treated for optimal cell attachment, with canted neck and with vented cap from Corning Inc. Prior passaging, the culture media, 1 x PBS and trypsin-EDTA solution was pre-warmed to 37°C in a Fisher Scientific DMU19 Water Bath. For passaging, cells were washed twice with 1 ml of 1 x PBS solution per 10 cm2 growth area and detached by incubating at 37°C for 5-6 minutes in 0.4 ml of 0.1% (w/v) trypsin, 0.02% EDTA solution per 10 cm2 of cell growth area. Cell-trypsin solution was transferred to 15 or 50 ml conical polypropylene CentriStar™ centrifuge tubes containing 10 ml of culture medium with serum and centrifuged for 5 minutes at 150 g in a MSE Mistral 2000 centrifuge. The cell pellet was re-suspended in fresh culture media, transferred to new cell culture flasks and placed back in the cell culture incubator. Integra Pipetboys with 5 ml (734-1737), 10 ml (734-1738) and 25 ml (734-1739) polystyrene Stripettes attachments were used to handle liquid volumes in the order of millilitres. Microliter volumes were measured using 10 µl (S1120-3810), 200 µl (S1120-8810), 1,000 µl (S1126-7810) StartLab TipOne Filter Tips connected onto Eppendorf Research plus, adjustable-volume, single channel pipettes. For liquid disposal, a vacuum line was used which consisted of autoclaved glass

Pasteur pipettes (612-1701, VWR) and C-Flex tubing (190-947-001, Cole-Parmer), a Büchner flask containing Klorsept 87 solution (CLE1490, Scientific Laboratory Supplies Ltd.), a Whatman Vacu-Guard Filters (09-744-75, Thermo Fisher Scientific Inc.) and a central vacuum pump. Seventy percent ethanol was used to sterilize surfaces. Conical 15 ml (734-1867) or conical-skirted 50 ml (734-1876) polypropylene CentriStar tubes with high-density polyethylene caps were purchased from VWR. DMEM (D5546), L-Glutamine (G8540), EC growth factors (E2759), heparin (H0777), FCS (F7524), collagenase (C9891), gentamycin (G3632), amphotericin (A9528), HBSS (H9269), Penicillin-Streptomycin (P4333) and Trypsin-EDTA solution (T4049) were supplied by Sigma-Aldrich Corp. Dulbecco's phosphate buffered saline 10x (D1408) from Sigma-Aldrich Corp. was diluted 1:10 (v/v) in autoclaved Milli-Q water to obtain 1xPBS. Ethanol (20821.321) was ordered from VWR BDH Prolabo.

Freezing of mammalian cells

For storage, mammalian cells were frozen in liquid nitrogen. For this, cells were detached by washing twice with 1 x PBS, followed by trypsinization and cells were pelleted, as described above. The pellet was re-suspended in culture media with double the normal FBS concentration. A 10 µl aliquot of the cell suspension was mixed with 10 µl 0.4% (w/v) trypan blue solution and cells were counted using the Hawksley Improved Neubauer BS 748 haemocytometer. To the mother solution, another solution, which besides the double to normal FBS concentration also contained 20 % dimethyl sulfoxide (DMSO), was added drop-wise until the two solutions reached a volumetric ratio of 1:1. The final cell concentration was of 1 million cells per ml. One ml of this cell suspension was distributed per cryovial and cryovials were placed in the Thermo Fisher Scientific™ Mr. Frosty™ Freezing Container which contained 250 ml of isopropanol pre-chilled to 4°C. Mr. Frosty™ Freezing Container was then placed into the -80°C in a New Brunswick Scientific ultra-low temperature U 725-86 lab freezer for 24 hours and subsequently cryovials were transferred to the gas phase of liquid nitrogen. Since the DMSO solution was not sterile, the culture medium which contained 20% DMSO, and had double the normal FBS concentration, was filtered through two cellulose acetate membrane VWR filters connected in a series. The first filter had a pore diameter of 0.45 µm (514-0063), whereas the pore diameter of the second filter was 0.2 µm (514-0061). The liquid was pushed through these filters using a 50 ml syringe (613-4902) from VWR. Isopropanol (20842.323) was ordered from VWR BDH Prolabo. DMSO (D8418) and 0.4% (w/v) trypan blue (T8154) solutions were supplied by Sigma-Aldrich Corp. Fisherbrand polypropylene 2 ml cryovials (12567501) were acquired from Thermo Fisher Scientific Inc.

Thawing of mammalian cells

Cryovials of cells were removed from the liquid nitrogen tank or from the -80°C New Brunswick Scientific ultra-low temperature U 725-86 lab freezer and were immediately placed in a 100 ml beaker containing 37°C water, avoiding the water to reach the cap area of the cryovial in order to prevent contamination. As soon as the cell solution melted, it was transferred into a 50 ml conical-skirted polypropylene CentriStar™ capped tube which contained 10 ml culture media pre-heated to 37°C in order to dilute the DMSO. Cells were then pelleted by centrifuging for 5 minutes at 250 g in a MSE Mistral 2000 centrifuge. The supernatant which contained the DMSO was removed using the vacuum line. Cells were re-suspended in culture media, pre-warmed at 37°C and placed in a Rectangular Corning polystyrene cell culture flask with 25 cm^2 CellBIND cell growth area in 6 ml culture media. Cells were placed in 20 ml of culture media when seeding in a cell culture flask with 75 cm^2 CellBIND cell growth area. The day after seeding, cells were visualized under a under Leica DM IL LED–DFC295 or Leica DM IL LED–DFC290 inverted phase contrast microscopes and checked whether cells were adhering and presenting the morphology characteristic for healthy cells. At this time, the culture media was refreshed by aspirating the old media with

the vacuum line and adding the equivalent volume of fresh media pre-warmed at 37°C. Cells were placed back into cell culture incubator and cultured using the standard procedure from here onwards.

Electroporation transfection

For plasmid electroporation into mammalian cells, the Neon™ Transfection System from Life Technologies Inc. with 10 µl electroporation tips was used following the manufacturer's instructions. Briefly, 24 hours prior electroporation, cells were sub-cultured or fed with fresh culture medium; to obtain 80-90% cell confluency at the time of electroporation. To electroporate, cells were washed twice with PBS, trypsinized, pelleted, re-suspended in PBS and counted using the Hawksley Improved Neubauer BS 748 haemocytometer. The appropriate amount of cells (50,000 – 300,000 cells per electroporation reaction) was pelleted and resuspended in Resuspension Buffer R (proprietary content, provided with the Neon™ Transfection System) and 1 µl of plasmid solution (3-15 µg/µl DNA concentration) was added for every 9 µl of Buffer R solution. Next, the 10 µl electroporation Neon Tip was picked up with the Neon Pipette. After mixing, this cell-DNA mixture was aspirated in the 10 µl electroporation Neon Tip, avoiding air bubbles, and the Neon Tip – Neon Pipette assembly was inserted into the Neon Tube. The Neon Tube was pre-loaded with 3 ml of proprietary Electrolytic Buffer E and was pre-mounted on the Neon Pipette Station. Next, the cell type specific electroporation pulse was applied from the Neon Device which was connected to the Neon Pipette Station. The Neon Pipette together with the cell-DNA solution containing Neon Tip was removed from the Neon Tube and the 10 µl content of the Neon Tip was emptied and mixed into antibiotic-free culture medium pre-incubated in the cell culture incubator (to reach optimal temperature and CO_2 content). The multi well plate or culture flask containing the electroporate cells was placed back into the cell culture incubator and 4-16 hours after electroporation the culture medium was renewed. The wells of multi well plates were pre-coated with 1% gelatine solution by incubating 30 minutes in cell culture incubators prior usage for cell culturing purposes. The volume of cell culture solution and of the 1% (w/v) gelatine solution in wells of multi well plates was 2, 1, 0.5 and 0.25 ml for 6-well, 12-well, 24-well and 48-well plates, respectively.

To electroporate cells for flow experiments, cells were seeded in a 0.5 x 5 cm well consisting of a glass slide clamped together with a Polydimethylsiloxane (PDMS) part. Prior seeding electroporated cells, this well was coated with 150 µL 1mg/ml fibronectin solution by incubating in the cell culture incubator for 1 hour and then 800 µl of culture media was placed in this well to incubate in the cell culture incubator for 2 hours.

Pulse voltage (500 – 1500 V), pulse duration (5 – 500 ms), number of pulse (1 – 20) and cell electroporation density (50,000 – 300,000 cells per 10 µl) were optimized individually for each cell type. Six-well (734-1596), 12-well (734-1597), 24-well (734-1606) and 48-well (734-1607) Corning plates with surfaces treated for tissue culture were ordered from VWR. The Neon™ Transfection System 10 µL Kit (MPK1096) and Fibronectin from bovine plasma (33010-018) was ordered from Life Technologies Inc. Gelatin from bovine skin (G9382) and Fibronectin from bovine plasma (F1141) was ordered from Sigma-Aldrich Corp. Fibronectin from bovine plasma (341631) was also ordered from Merck Chemicals KGaA., Darmstadt, Germany.

Mammalian antibiotic selection

To determine the optimal neomycin (G 418) antibiotic concentration for selection, EA.hy926 cells were seeded in 12-well plates, pre-coated with 1% (w/v) gelatine, at a density of 3,100 cells/cm^2. Twenty-four hours after seeding, the following G 418 concentrations were added to the wells of the 12-well plate: 0, 100, 200, 300, 400, 500, 600, 700, 800, 1,000, 1,200 and

1,500 µg/ml. The media was replaced every 2-3 days with media containing the corresponding antibiotic concentrations and the selection was carried out for a period of 7 days. A similar titration experiment was also carried out for the Hygromycin B antibiotic. The antibiotic concentration 100 µg/ml above the lowest mammalian selection antibiotic concentrations which killed all the EA.hy926 cells were used in subsequent selection experiments. To select EA.hy926 cells that contain p10 and p12 (**Table S1**), EA.hy926 cells were co-transfected with p10, p12 and the Linear Hygromycin Marker, the molar ration of the Linear Hygromycin Marker to plasmids was 1:20. Twenty-four hours after transfection, 400 µg/ml of Hygromycin B and 500 µg/ml of G 418 antibiotics were added and re-added in fresh culture media every 2 – 3 days for a period of 6-7 days. Following the same procedure, 100 µg/ml of Hygromycin B and G 418 antibiotic concentrations were also used for selection.

G 418 antibiotic was also titrated on HMEC-1 cells to determine the optimal G 418 concentration for a 2 days selection. For this titration, HMEC-1 cells were seeded in a 24-well plate, pre-coated with 1% (w/v) gelatine, at a density of 5,200 cells/cm^2 and 24 hours after seeding, G 418 antibiotic was added in the 0 – 2,000 µg/ml concentration range. The antibiotic concentration 100 µg/ml above the lowest G 418 concentration that killed the cells was used to select HMEC-1 cells transfected with the p10 plasmid in a 2 days selection experiment.

Optimizing the mammalian antibiotic selection procedure revealed that the highest percentage of cells with responsive gene network was achieved when HMEC-1 cells were electroporated with the p10 plasmid, 24 hours later 200 µg/ml of G 418 was added, 48 hours after G 418 addition the selection pressure was removed, 24 hours after this cells were electroporated with the p12 plasmid and 24 hours after the second electroporation the gene network was induced. Ten minutes after the second electroporation, the gene network was inhibited with doxycycline and doxycycline was removed 2 minutes before inducting the gene network. Linear Hygromycin Marker (631625), comprising of the marker gene, an SV40 promoter, and the SV40 polyadenylation signal, were ordered from Clontech. G 418 disulfate salt (A1720-1G) was ordered from Sigma Aldrich Corp. Hygromycin B (10687-010) purchased from Life Technologies Inc.

Induction and inhibition of the gene network

To induce the expressed gene network, 6 hours after electroporation, 2 µM of [Hyp^3]-Bradykinin and 2 µM of Bradykinin [20-23] was added to the culture and the following day the cells were imaged. To enhance the induction, the same amount of [Hyp^3]-Bradykinin and Bradykinin was re-added every 6 – 12 hours after the first addition, knowing that Bradykinin has a half-life in the order of seconds to minutes [24-28]. For induction, shear stress was also applied by pipetting using a 1,000 µl Eppendorf Research plus, adjustable-volume, single channel pipette with 1,000 µl StartLab TipOne Filter Tips.

To induce the gene network by applying well controlled shear stress, flow experiments were carried out using both conventional and in-house developed flow setups (see below).

To inhibit the gene network at the shear stress sensor level, 20–100 nM of the HOE 140 selective B2 Bradykinin receptor antagonist was added 1 – 24 hours after electroporation [29-31]. To inhibit the gene network at the transcription factor (tTA) level, 20 ng/ml doxycycline was used [32,33].

Bradykinin acetate salt (B3259-1MG), [Hyp^3]-Bradykinin (B7775-1MG), HOE 140 (H157-250UG) and Doxycycline hyclate (D9891-1G) were purchased from Sigma-Aldrich Corp.

Design of linearly increasing shear stress inducing flow channel and validation by computational fluid dynamics

A 3-Dimensional Computer Aided Design (3D CAD) model of a 0.5 cm wide, 5 cm long flow channel with a height varying along the channel length described by a square root function, was generated in SolidWorks 2012 version 20.0. The shear stress at the bottom wall of the channel (channel floor) in the resulting model was calculated using the Flow Simulation 2012 Computational Fluid Dynamics package in SolidWorks. For the flow simulation, the interior of the channel was selected as the computational flow domain. For boundary conditions, in the channel model there was one inlet, one outlet and all other boundaries were considered to be rigid and impermeable walls. The inlet flow rate was set to 5 ml/min and at the outlet standard atmospheric pressure (1 atm = 101325 Pa) was modelled. Water at 20.05°C was used as the computational fluid; water was a close approximation of mammalian cell culture medium that was used in flow experiments in the actual experimental setup. For meshing, advanced geometry resolution and result resolution of 6 (out of 8, default value being 3) was used. Mesh refinement was automatically done by SolidWorks Flow Simulation package by dividing the computational domain into elementary rectangular volumes and subdividing these volumes into further elementary rectangular volumes in accordance with the results and geometry resolution settings. For validation, obtained flow velocities were compared to analytically calculated flow velocities.

Manufacturing of linearly increasing shear stress inducing flow channel

To manufacture the bottomless flow channel with the desired geometry, both 3D printing (also known as rapid prototyping) and casting were employed. Rapid prototyping and manufacturing of metal parts was carried out at the Mechanical Instrumentation Workshop, Department of Physics, Imperial College London. For 3D printing, FullCure720 rapid prototype resin was printed using an Objet Eden250 Professional 3D Printer in high quality printing mode which yields a resolution of 16 µm for the height of flow channel. For casting, both polydimethylsiloxane (PDMS) and thermosetting polyester (TPE) were separately poured onto metal mould patterns contained inside a petri dish. The fabrication of the metal mould parts is described in the section below. To cast the PDMS, first the elastomer (monomer, base) was mixed with the curing agent at a 10:1 volumetric ratio. This solution was poured on top of the mould and was degassed in a vacuum chamber to remove air bubbles. Next, the degassed PDMS was cured at 60°C for 3 hours in an FMA-275 Grieve oven. After curing, the PDMS was detached from the metal mould to obtain the bottomless flow channel. The geometry of obtained flow channels was verified with a Xyris 4000 Confocal Laser Surface Profiler from Taicaan Technologies. For TPE casting, 1 part of Methyl ethyl ketone peroxide catalyst was added to 100 parts of polyester resin, was mixed and briefly degased under vacuum and was cured at room temperature for 36 hours. After curing, the TPE was removed from the metal mould to obtain the bottomless flow channel. Fullcure720 (RGD720) rapid prototype resin was acquired from Stratasys Ltd., Edina, MN, USA. SYLGARD 184 silicone elastomer PDMS kit was supplied by Dow Corning Corporation (MI, USA). Water clear casting TPE resin was provided by CFSNET Ltd. Redruth, Cornwall, UK.

Manufacturing of metal mould parts for casting the linearly increasing shear stress inducing flow channel

The curved channel height profile of the metal wedge mould was generated by high precision wire-cut electric discharge machining (EDM) using a Fanuc Robocut α-oic wire-cut electric discharge machine. At both ends of this wedge, round holes were drilled using a Bridgeport EZ vertical milling machine. Into these holes long cylinders were fixed using epoxy glue, to obtain inlets and outlets for flow in the casted channel. These long cylinders were cut using an Emcomat 14-D Lathe machine. The wedge with cylinders attached was then fitted into a base part into a rectangular hole cut with wire-cut EDM using the Fanuc Robocut α-oic machine. Exterior sides of this base part were cut using a Bridgeport EZ

vertical milling machine. The material of metal parts was H30 6082T651 aluminium alloy acquired from Smiths Metal Centres Ltd., Biggleswade, UK. For metal-to-metal gluing, Loctite Epoxy Metal from Loctite Düsseldorf, Germany was used.

Manufacturing of the cell seeding well

To facilitate cell seeding a well was also created. The bottom of the well consisted of a 7.5 x 2.5 cm standard plain microscopy glass slide. The 5.5 x 0.5 cm rectangular well was cut into the centre of a 7.2 cm long, 2.3 cm wide and 1 cm thick PDMS cuboid plate. By placing this PDMS piece on top of the glass slide, a 0.5 cm deep, 5.5 x 0.5 cm rectangular well was obtained. This PDMS part was obtained by casting PDMS on a metal mould. The metal mould consisted of the wedge described in the section above, without placing this wedge into the 0.5 cm thick base part. This wedge was placed on the centre of a 7.5 x 2.5 cm standard plain microscopy glass inside a petri dish. For casting, first 9 volume parts of the monomer base were mixed with 1 volume part of the curing agent. This solution was poured onto the metal wedge mould inside the petri dish and PDMS was degassed in a vacuum chamber to remove air bubbles. Next, the degassed PDMS was cured at 60°C for 3 hours in an FMA-275 Grieve oven. After curing, the PDMS was detached from the metal mould and exterior of the PDMS well part was cut and rectified with a SM0510 surgical scalpel blade from Swann-Morton Ltd., Sheffield, UK. Shandon™ 1.0-1.2 mm thick, 76 x 26 mm plain microscopy glass slides (6776111) were purchased from Thermo Fisher Scientific Inc. Corning plain microscope glass slides (CLS294775X25-72EA) were ordered from Sigma-Aldrich Corp.

Manufacturing of commentary equipment for the flow device

Complementary metal parts for clamping the bottomless flow channel or seeding well to the glass slide, and for visualizing cells, cultured on the glass, under standard microscopes, were cut using the Bridgeport EZ vertical milling machine. To clamp the metal plates together with the bottomless flow channel and glass slide in between, standard metric M4 steel screws with M4 steel washers were screwed from the top of the device into the base plate. Holes for screws were drilled using the Bridgeport EZ vertical milling machine and the screw threads in the base plate were generated using a M4 x 0.70 threading and drilling hand-tool from Wiseman Threading Tools Ltd., Redditch, UK. The material of all generated metal parts except the screws and washers was H30 6082T651 aluminium alloy provided by Smiths Metal Centres Ltd., Biggleswade, UK. Standard metric M4 coarse thread high tensile steel screws and M4 stainless steel washers were provided by TR Fastenings Ltd., Uckfield, UK.

Flow experiments

To expose cells to well controlled levels of shear stress, flow experiments were carried out. The flow setup consisted of a peristaltic pump, a bubble catcher media reservoir, platinum-cured silicone tubing and a flow channel. The peristaltic pump was either a Cobe precision peristaltic pump (03600-001) from COBE Laboratories, Inc., Lakewoor, CO. USA or a Perista Pump (SJ-1220) with 4 channels from Atto Bioinstrument Corp., Japan. Two different high temperature resistant, inert media reservoirs were used. The first one was a 150 ml glass chamber with a loosely fitted lid modified with a media inlet and with an outlet at the bottom of the chamber. The second reservoir was a 500 mL Schott glass bottle with a modified cap. The screw-on polypropylene cap was perforated to fit three tubes: media inlet, media outlet and gas exchange (fitted with a 0.22 µm hydrophobic filter). The flow channel was either a conventional µ-Slide I $^{0.2}$ Luer (80166 or 80161), µ-Slide I $^{0.4}$ Luer (80176 or

80171), µ-Slide I $^{0.6}$ Luer (80186), µ-Slide I $^{0.8}$ Luer (80196 or 80191) channel from ibidi GmbH., Martinsried, Germany or the in-house designed flow channel (cf. the following subsection). For the pump head, a 36 cm long platinum-cured silicone tubing (WZ-95802-04) of 3 mm inner diameter and 4.8 mm outer diameter from Cole Parmer was used. The rest of the components of the flow setup were connected by platinum-cured silicone tubing (WZ-95802-03) of 2.29 mm inner diameter and 4 mm outer diameter from Cole Parmer. The tubing was connected to the flow channel using Luer to 200 series barb elbow connectors with 3.2 mm inner diameter from The West Group Ltd., Waterlooville, UK. The pump head tubing was connected to the rest of the tubing using straight through connector with classic series barbs with 3.2 mm inner diameter from The West Group Ltd. The flow setup was sterilized by autoclaving at 135 °C for 5 minutes in a MLS-3751L Sanyo autoclave. Seventy ml of culture media was added to the media reservoir and the flow setup was placed in a cell culture incubator for 2 hours to equilibrate the gas concentrations and temperature before starting the flow experiment. For flow experiments using ibidi µ-Slide I Luer Family channels, channels were coated with 1 mg/ml fibronectin or 1% (w/v) gelatine. In case of channels with tissue culture treated ibiTreat hydrophilic surfaces, no further coating was applied. Next, cells were seeded at a density of 40000 cells/cm^2. Twenty-two hours after cell seeding, 60–80 ml culture media was placed in the bubble-catcher media reservoir of the flow setup and this setup was placed inside the cell culture incubator for two hours in order to allow the culture medium to reach the temperature and gas content optimal for cell experiments. Twenty-four hours after cell seeding, the tubing of the pre-equilibrated flow setup was connected to the cell containing flow channel, avoiding air bubbles. The pump head tubing was coupled to the pump and the flow experiment was started by priming cells for 2 hours at 0.2–0.5 Pa. Over the course of the following hour, the flow rate was gradually increased to yield the experimental 1.5-2 Pa shear stress for 24 hours. When the Atto Perista Pump was used the entire setup, including the pump was placed inside the cell culture incubator. In case of the Cobe pump, the flow setup was placed inside the incubator but the pump stayed outside the incubator. The culture media was circulating from the media reservoir, through the flow channel to the pump and back to the media reservoir.

Flow experiments carried out in the in-house designed flow device are described in below.

Operation of the linearly increasing shear stress inducing microfluidics device

The flow setup was similar to the one described in the previous section, with the exception of the flow channel and the complementary clamp-microscope holder. The PDMS flow channel, the PDMS seeding well, the complementary clamp-microscope holder with screws, the bubble catcher media reservoir and platinum-cured silicone tubing was sterilized by autoclaving at 135 °C for 5 minutes in a MLS-3751L Sanyo autoclave. The 7.5 x 2.5 cm standard plain microscopy glass slide was sterilized by autoclaving. Working under sterile conditions, the glass slide was placed in the metal holder, the PDMS seeding well was placed atop of the glass slide, the top metal slide was placed over the PDMS well and the two metal parts was screwed together applying even torque. 150 µl of 1 mg/ml fibronectin solution was added to the obtained seeding well. The device was placed in a 1000 µl pipette tip box modified to accommodate the device and this box was placed in the cell culture incubator at 37 °C for 1 hour. After coating, the fibronectin solution was removed and cells were seeded at 40.000 cells/cm^2 density in 500 µl of culture media. Twenty-four hours after cell seeding, the device was disassembled and the PDMS seeding well was replaced by the bottomless PDMS flow channel, screws being again evenly torqued. The culture media for flow experiments was pre-equilibrated in the cell culture incubator for 2 hours to temperature and gas concentrations required for cell growth. The tubing of the media reservoir, containing the pre-equilibrated media was connected to the cell containing flow channel, avoiding air bubbles. The flow setup was placed in the cell culture incubator and the pump head tubing was connected to the peristaltic pump. The Cobe precision peristaltic pump remained outside the incubator.

The flow experiment was started by priming cells for 1 hour at 0 – 0.5 Pa shear stress, then the flow rate was gradually increased to yield the experimental 0.2 – 10 Pa shear over the following 2 hours and then maintained at the experimental level for 24, 36 or 48 hours.

In all flow experiments the culture media was flowing from the media reservoir, through the flow channel to the pump and back to the media reservoir. At the end of the flow experiment, the pump was stopped, the pump was separated from the rest of the flow setup, the flow channel was dismounted and the slide was visualised under a microscope.

Cell staining and fixation

At the end of the flow experiments, the flow setup was disassembled. In case of the in-house designed flow channel, the glass slide with the cell monolayer was placed in a round petri dish (101VBLUE) with a diameter of 9 cm from Sterilin Ltd., Newport, UK. To assess cell viability, propidium iodide (P3566) from Life Technologies Inc. was used to stain the DNA of dead cells. The nuclei of all cells (dead and alive) were stained with Hoechst 33342 (H3570) from Life Technologies Inc. EA.hy926 cells were stained with 0.5 µg/ml propidium iodide in DMEM solution by incubating at room temperature for 30 minutes. HeLa and EA.hy926 cells were stained with 2 µg/ml Hoechst 33342 DMEM solution by incubating at room temperature for 5 minutes. HMEC-1 cells were stained with final concentration of 1 µg/ml propidium iodide and 2 µg/ml Hoechst 33342 solutions in DMEM by 10 minutes incubation. Following staining, cells were washed twice with PBS and fixed with 4% (paraformaldehyde) PFA by incubation at room temperature in a fume hood for 15 minutes, then cells were washed twice again with PBS and stored at 4°C for subsequent imaging.

Microscopy imaging

Cells were visualized under Leica DM IL LED–DFC295 or Leica DM IL LED–DFC290 inverted phase contrast microscopes. Cells were imaged using a Hamamatsu ORCA-ER camera coupled to a Zeiss Axiovert 200 inverted fluorescent microscope with a fully motorised stage, controlled by Improvision Volocity acquisition software. For imaging, the brightfield Köhler illumination was first adjusted and the Zeiss EC Plan-Neofluar 10x 0.30 Ph1 objective was used with the phase contrast 1 channel (Ph1), Zeiss Filter Set 01 (DAPI), Zeiss Filter Set 15 (TRITC) and Zeiss Filter Set 10 (FITC) in combination with a mercury fluorescence lamp. To capture the surface of the entire cell monolayer or slide, the microscope was programmed for tile acquisition. For this, the microscope stage was first calibrated, the region of interest (ROI) was defined, a focus map was created using 5-10 randomly selected points per cm^2 of the ROI, and finally the tile acquisition protocol was stared using 10-20% overlap between tiles and following the created focus map to keep the specimen in focus.

Image processing for determining cell viability and transfection efficiency

To count cells, the acquired microscopy images were processed in ImageJ 1.47 [34,35] . As a first step, images were calibrated (by correlating pixels to micrometres) by drawing a line (measured in pixels) that had the same length as the scale-bar (measured in µm). Then, the pixel/µm ratio was determined using the Set Scale function in imageJ. Next, the size (area) range of cells to be counted was determined by measuring a very small cell and setting its dimensions as the lower limit of the range, the upper limit was set to infinity. For the actual cell counting, the colour threshold was adjusted until the background noise disappeared, images were converted to binary, touching cells were separated using the watershed segmentation algorithm, and finally cells were counted with analyse particles command. In the analyse particles function, the circularity was set from 0.10 – 1.00 and the size of the

cells to be counted was set to the predefined value range. To automate the process, a macro script was created and applied for the steps following colour thresholding.

Counting Hoechst 33342 stained blue fluorescent nuclei gave the total number of cells, propidium iodide stained red fluorescent cells showed the number of dead cells, and cells turned green fluorescent due to eGFP or MGFP expression represented the positive cells. The percentage of green fluorescent cells and cell viability was calculated from fluorescent images recorded on the same cell population using Equation 2.1 and Equation 2.2, respectively.

$$Gp = \frac{\mathrm{Gn}}{\mathrm{Bn}} \times 100 \ \ [\%]$$

Equation 2.1. Calculation of the percentage of green fluorescent cells from fluorescent microscopy images. Where: Gp: Percentage of green fluorescent cells. Gn: Number of green fluorescent cells (eGFP or GFP expressing cells) Bn: Number of blue fluorescent cells; total number of cells, including dead and living cells (Hoechst 33342 stained cells).

$$\mathrm{Cv} = 100 - \frac{\mathrm{Rn}}{\mathrm{Bn}} \times 100 \ \ [\%]$$

Equation 2.2. Calculation of cell viability from fluorescent microscopy images. Where:

Cv: Cell viability, in percentage. Rn: Number of red fluorescent cells; number of dead cells (propidium iodide stained cells). Bn: Number of blue fluorescent cells; total number of cells, including dead and live cells (Hoechst 33342 stained cells).

Additionally, mean fluorescence intensities were determined in Volocity Image Analysis Software. Statistical significance of mean fluorescence intensities was calculated using 2 way ANOVA in SigmaPlot v12 from Systat Software Inc.

Supplementary Table

Table S1. Mammalian expression DNA plasmids

Plasmid name	Gene content	Plasmid backbone	Plasmid size [kbp]	Antibiotic marker	Source
p1	α2A-AR-AVPR2tail-TCS-tTA	pCI (Promega)	6.5	Ampicillin	Dr. Barnea*
p2	βArrestin2-TEV	pcDNA3	7.4	Ampicillin	Dr. Barnea*
p3	Luciferase tango reporter	pUHC 13-3	5.5	Ampicillin	Dr. Barnea*
p4	BDKRB2	pBSII SK(+)	4	Ampicillin	Eurofins
p5	eGFP	pCR2.1	4.7	Ampicillin	Eurofins
p6	BDKRB2-AVPR2tail-TCS-tTA	pCI (Promega)	6.2	Ampicillin	In-house
p7	eGFP reporter	pUHC 13-3	4.9	Ampicillin	In-house

p8	Constitutive Monster GFP	phMGFP	4.7	Ampicillin	Promega
p9	Bicistronic plasmid	pIRES	6.1	Ampicillin	Clontech
p10	BDKRB2-AVPR2tail-TCS-tTA-IRES-βArrestin2-TEV	pIRES	10.3	Ampicillin, neomycin (G 418)	In-house
p11	TRE3G promoter	pTRE3G	3.4	Ampicillin	Clontech
p12	eGFP reporter	pTRE3G	4.1	Ampicillin	Dr. Wilson$
p13	hKLF2-GFP	pBRYCAG-floxStop dsRED-IresPuro	9.8	Ampicillin, Puromycin	Addgene
p14	hKLF2-GFP reporter	pTRE3G	4.4	Ampicillin	In-house

Legend: α2A-AR - human α2A-adrenergic receptor; AVPR2tail - last 29 amino acids of human arginine vasopressin receptor 2; TCS - tobacco etch virus protease (TEV) cleavage site; tTA - tetracycline-controlled transcriptional trans-activator; α2C-AR - human a2C-adrenergic receptor; β1-AR - human b1-adrenergic receptor; β2-AR - human b2-adrenergic receptor; βArrestin2 - human beta-arrestin2; TEV - catalytic domain tobacco etch virus NIa protease (amino acids 189-424 of the mature NIa protease); Luciferase – firefly luciferase, BDKRB2– human bradykinin receptor B2; eGFP – enhanced green fluorescent protein; Monster GFP – monster green fluorescent protein; IRES – Internal ribosome entry site; hKLF2 – human Krüppel-like factor 2.

* Courtesy of Dr. Gilad Barnea from Brown University, USA, tango assay developer

$ Courtesy of Dr. Miranda SC Wilson and Dr. Adolfo Saiardi from the MRC Laboratory for Molecular Cell Biology, University College London, UK.

Supplementary Figures

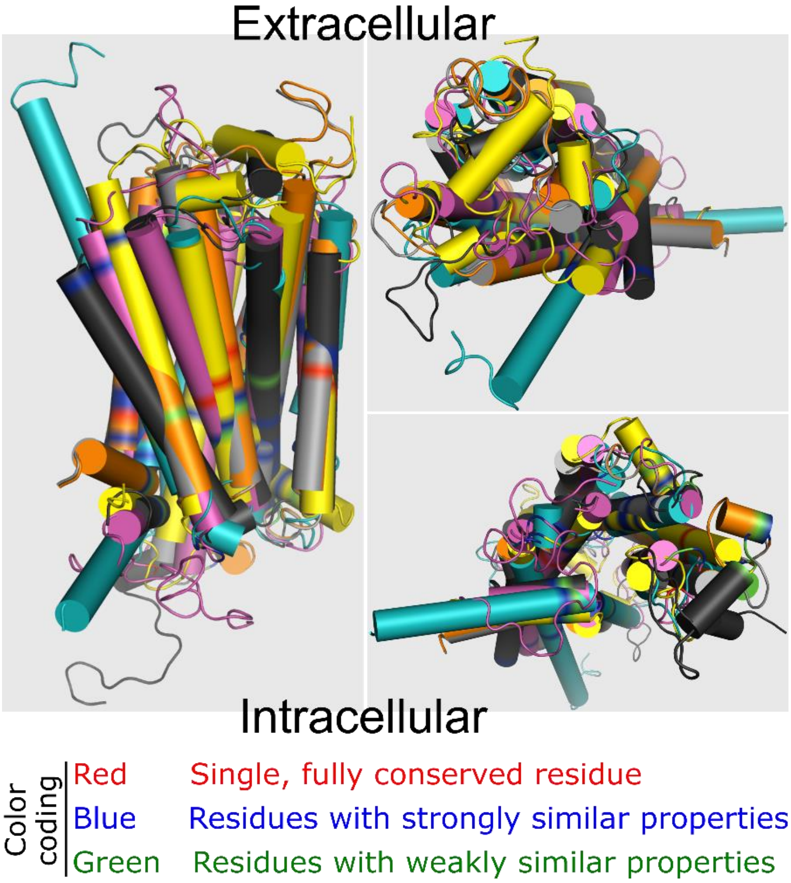

Fig. S1. Prediction of shear stress sensitive segments in the Bradykinin B2 GPCR, based on sequence similarities with 6 presumed shear stress sensitive GPCRs: Bradykinin receptor B2 (BDKRB2) [36], Formyl peptide receptor 1 (FPR1) [37], Parathyroid hormone receptor 1 (PTH1R) [38], Dopamine receptor D5 (DR5) [39], Sphingosine-1-phosphate receptor-1 (S1PR1) [40], G protein-coupled estrogen receptor 1 (GPER1) [41,42]. The above 6 GPCR amino acid sequences were obtained from UniProt [3], NCBI Protein [4] and RCSB PDB [5] databases. These 6 GPCR sequences were aligned in Clustal Omega [7,8]. Consensus colors red, blue and green were used to indicate residues that are fully conserved, present strongly-similar, and weakly-similar properties, respectively. Protein topologies were modeled with TMHMM [9,10] and Phobius [11,12]. The tertiary structure of the 6 shear stress sensitive GPCRs was predicted with Phyre [13]. Obtained tertiary structured were aligned in PyMOL [14] and sequence similarities were marked using the color codes described above. Sequence similarities between the 6 shear stress sensitive GPCRs could be responsible for the shear stress sensitive function in these GPCRs. These potential shear stress sensitive segments occur mostly thought the transmembrane helixes. Therefore, the N- and C terminal sequences are potentially not responsible for the shear stress sensing functionality. In consequence, the N- and C terminal sequences could presumably be altered without deleting the shear stress sensing functionality from these GPCRs.

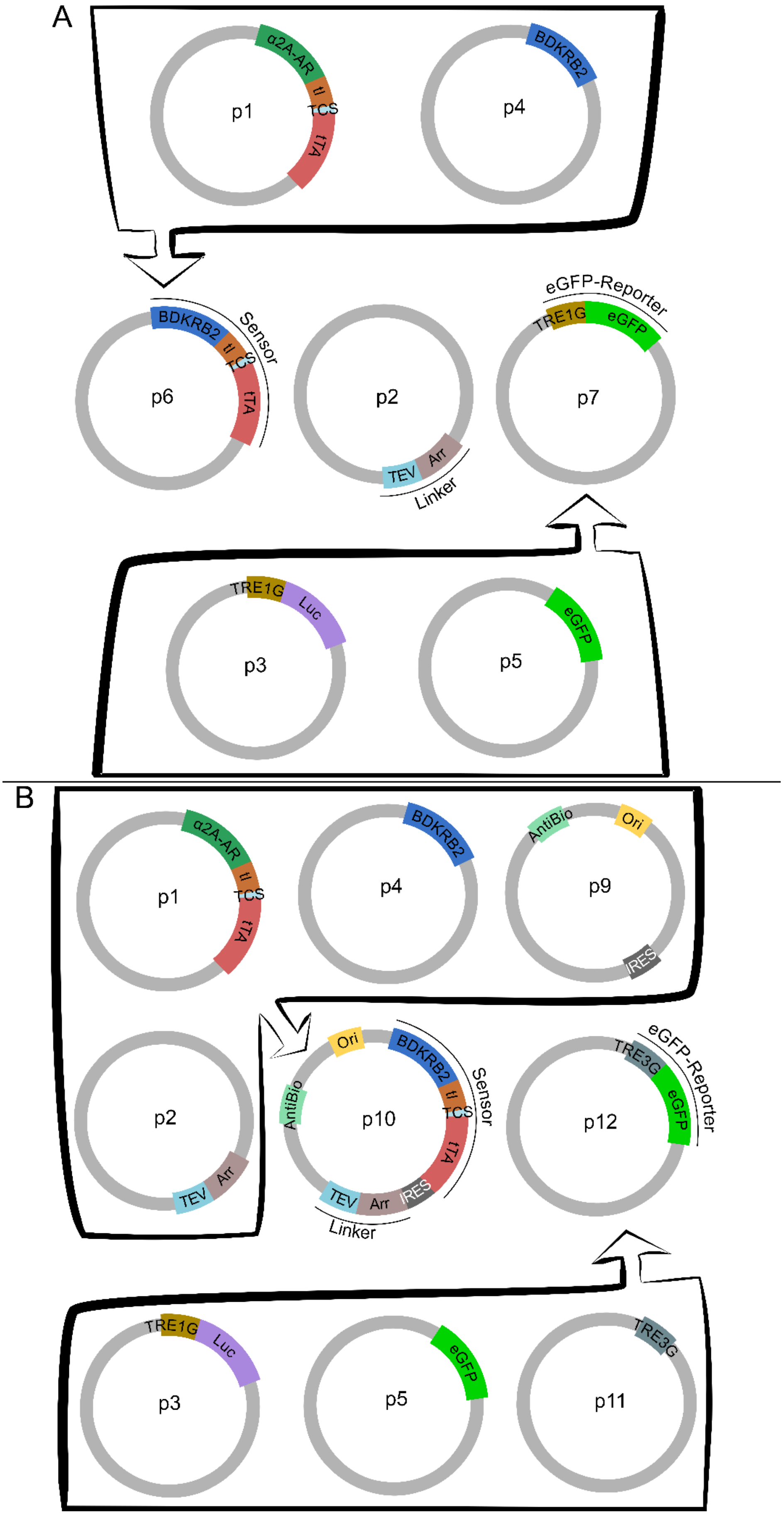
A
p1
α2A-AR
tl
TCS
tTA
p4
BDKRB2
p6
Sensor
p2
TEV
Arr
Linker
p7
eGFP-Reporter
TRE1G
eGFP
p3
Luc
p5
B
p9
AntiBio
Ori
IRES
p10
p12
TRE3G
p11

Fig. S2. Construction of the shear stress sensing gene network encoding plasmid. (A) Simplified representation of plasmids elements for building the first gene network version encoded on 3 plasmids. Illustrated DNA sequences from plasmids p1, and p4 were combined to obtain plasmid p6, which encodes the sensor module of the gene network. Plasmid 6 contains the backbone of p1. Illustrated DNA sequences from plasmids p3 and p5 were used to build plasmid p7 which encodes the reporter module of the gene network. Plasmid 7 contains the backbone of p3. (B) Simplified representation of plasmids elements for building the second gene network version encoded on 2 plasmids. Illustrated DNA sequences from plasmids p1, p2, p4 and p9 were combined to form plasmid p10 which encodes the sensor and linker modules of the gene network. p10 contains the backbone of p9. Illustrated DNA sequences from plasmids p3, p5 and p11 were used to build plasmid p12 which encodes the reporter module of the gene network. p12 contains the backbone of plasmid 11. For additional details of plasmids cf. Table S1. Abbreviation legend: Legend: α2A-AR - human α2A-adrenergic receptor; tl - last 29 amino acids of human arginine vasopressin receptor 2; TCS - tobacco etch virus protease (TEV) cleavage site; tTA - tetracycline-controlled transcriptional trans-activator; Arr - human β-arrestin2; TEV - catalytic domain tobacco etch virus NIa protease (amino acids 189-424 of the mature NIa protease); Luc – firefly luciferase, BDKRB2– human bradykinin receptor B2; eGFP – enhanced green fluorescent protein; IRES – internal ribosome entry site; TRE1G – 1st generation tetracycline responsive promoter; TRE3G – 3rd generation tetracycline responsive promoter; Ori – Simian virus 40 origin for replication in mammalian cells; AntiBio – neomycin resistance gene for mammalian selection.

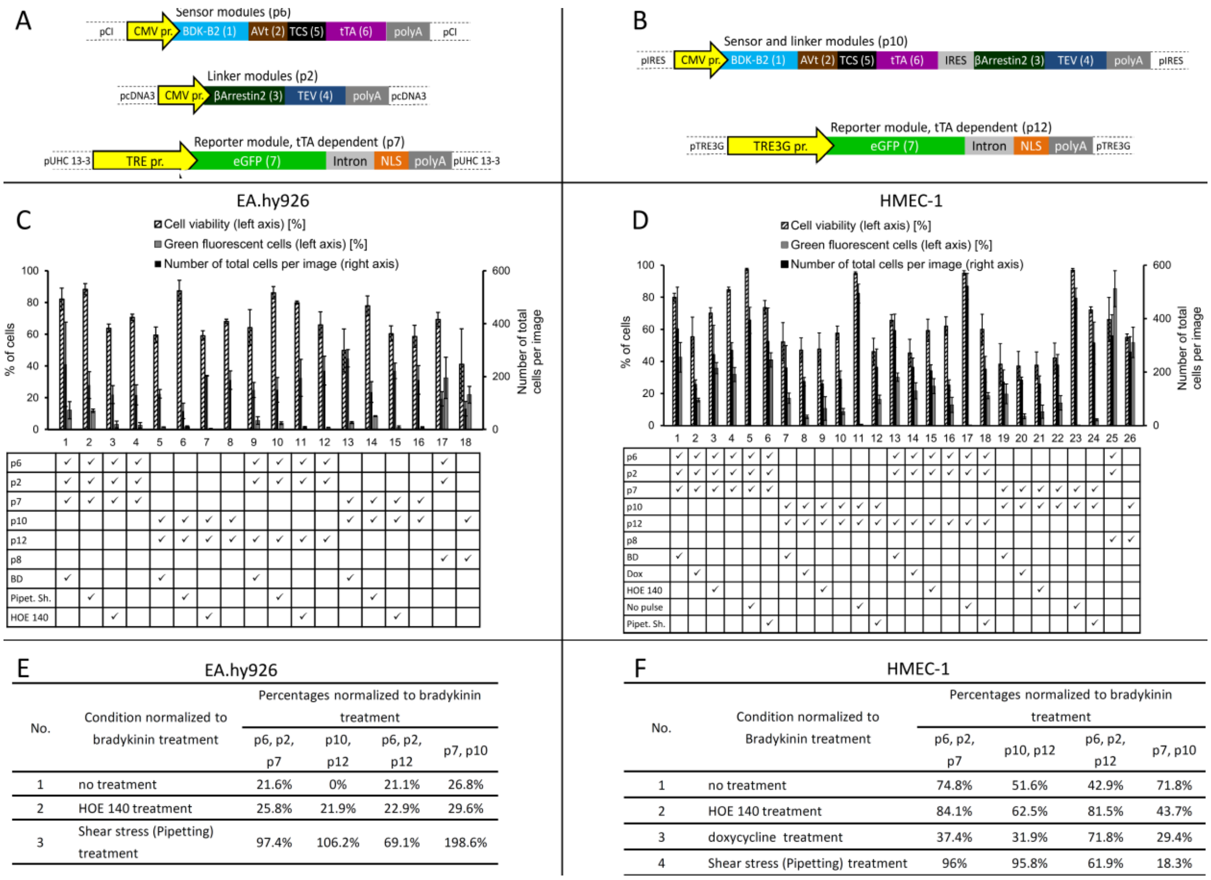

E

EA.hy926

No.	Condition normalized to bradykinin treatment	Percentages normalized to bradykinin treatment			
		p6, p2, p7	p10, p12	p6, p2, p12	p7, p10
1	no treatment	21.6%	0%	21.1%	26.8%
2	HOE 140 treatment	25.8%	21.9%	22.9%	29.6%
3	Shear stress (Pipetting) treatment	97.4%	106.2%	69.1%	198.6%

F

HMEC-1

No.	Condition normalized to Bradykinin treatment	Percentages normalized to bradykinin treatment			
		p6, p2, p7	p10, p12	p6, p2, p12	p7, p10
1	no treatment	74.8%	51.6%	42.9%	71.8%
2	HOE 140 treatment	84.1%	62.5%	81.5%	43.7%
3	doxycycline treatment	37.4%	31.9%	71.8%	29.4%
4	Shear stress (Pipetting) treatment	96%	95.8%	61.9%	18.3%

Fig. S3. Evaluating the performance of the two gene network versions. (A) Schematic representation of the plasmids encoding the gene network expressed from 3 plasmids. The sensor module is expressed from p6, the linker module is contained on p2, and p7 encodes the reporter gene. Additional abbreviations: human cytomegalovirus (CMV) immediate early constitutive promoter (CMV pr.), Simian virus 40 polyadenylation signal (polyA), Tet-responsive promoter (TRE pr.), nuclear localization signal of Simian virus 40 large T antigen (NLS). pCI, pcDNA3, pUHC 13-3 are plasmid backbones. (B) Schematic representation of the plasmids encoding the redesigned gene network expressed from 2 plasmids. The sensor and linker are on the bicistronic plasmid p10 and the reporter module is contained on plasmid p12. Abbreviations and colour codes correspond to part A above. Additional abbreviations: encephalomyocarditis virus internal ribosome entry site (IRES), 3rd-generation Tet-responsive promoter (TRE3G pr.). pIRES and pTRE3G are plasmid backbones. (C) Comparison between the performances of the two gene circuit versions (part A and B from above), between the performance of gene network components (reporter plasmid and signalling cascade), and between relative transfection efficiencies and gene expression levels in EA.hy926 cells. EA.hy926 cells were electroporated using optimized 900 V 30 ms double pulses and all experimental conditions were kept strictly identical between the 18 conditions, with the exception of parameters specified in the table below the graph. These varying parameters include: plasmids, and treatment conditions (shear stress induced by pipetting for 1 minute, 2 µM bradykinin, 100 nM HOE 140, or without treatment). Cells were stained with Hoechst 33342 and propidium iodide and imaged 48 hours transfection. Images were processed in imageJ, determined cell viabilities and transfection efficiencies are plotted on the left Y-axis and the total number of cells per image is plotted on the right Y-axis. Error bars represent standard deviation, N=3. (D) Comparing performances of gene circuit versions and components under various activating and inhibiting conditions, as well as comparing transfection efficiencies and expression levels in HMEC-1 cells. HMEC-1 cells were electroporated using optimized 1200 V 30 ms double pulses and all experimental conditions were kept strictly identical, except plasmids and treatment conditions (shear stress induced by pipetting for 1 minute, 2 µM bradykinin, 100 nM HOE 140, 20 ng/ml doxycycline, or no treatment) as specified in the table below the graph. Non-electroporated cells were also included as an additional negative control condition. Cells were stained with Hoechst 33342 and propidium iodide and imaged 48 hours transfection. Images were processed in imageJ, determined cell viabilities and transfection efficiencies are plotted on the left Y-axis and the total number of cells per image is plotted on the right Y-axis. Error bars represent standard deviation, N=4–6. (E) Green fluorescent cell percentages from no treatment, HOE 140 and shear stress treatment conditions normalized to green fluorescent cell percentages from the bradykinin treatment condition (values from part C). Normalized no treatment and HOE 140 treatment conditions show the leakiness of the gene network, which is the inverse of fold activation (responsiveness). (F) Green fluorescent cell percentages from no treatment, HOE 140 treatment, doxycycline treatment and shear stress treatment conditions normalized to bradykinin treatment green fluorescent cell percentages (values from part D). Normalized no treatment, doxycycline and HOE 140 treatment conditions show the leakiness of the gene network, which is the inverse of fold activation (responsiveness).

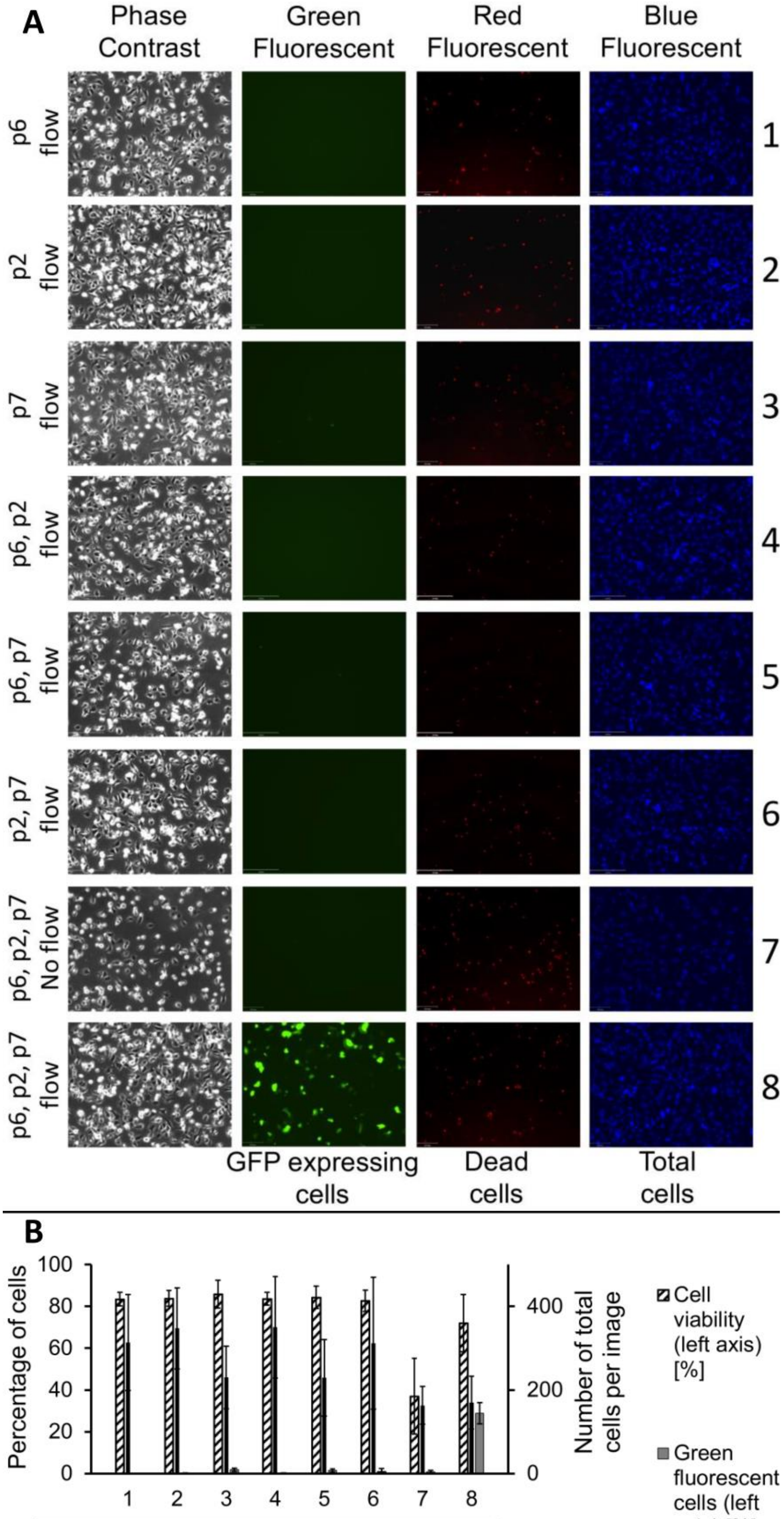

	1	2	3	4	5	6	7	8
p6	✓			✓	✓		✓	✓
p2		✓		✓		✓	✓	✓
p7			✓		✓	✓	✓	✓
Flow	✓	✓	✓	✓	✓	✓		✓
n	6	4	7	4	4	4	6	9

Fig. S4. Control experiments for determining the origin of GFP expression. EA.hy926 cells were electroporated with the plasmids indicated in the figure, using optimized 800 V, 30 ms double pulses. Next, these cells were exposed to 1.5–2 Pa shear stress 24 hours after electroporation for a period of 24 hours. 24 hours after the end of flow exposure, cells were stained with Hoechst 33342 and propidium iodide and then imaged. Alternatively, cells were also cultured under static conditions (without flow) and were stained and imaged 48 hours after electroporation as well. (A) Recorded phase contrast, green (GFP expressing cells), red (propidium iodide, dead cells) and blue (Hoechst 33342, total cells) fluorescent images are shown in the columns. Electroporated plasmids and shear stress exposure by fluid flow are specified for each row, and these conditions are numbered 1–9 on the right hand side of the images. 10X magnification. (B) Images were processed in ImageJ to count cells and determine the percentage of green fluorescent cells and cell viabilities. Percentages of green fluorescent cells and cell viabilities are plotted on the left Y-axis and the total number of cells per images is plotted on the right Y-axis. The conditions of the X-axis are described in the table below the graph and they correspond to conditions numbered 1–9 on the right hand side of the microscopy images (from part A). The number of analyzed images from separate experiments (n) is shown in the bottom row of the table. Error bars represent standard deviation.

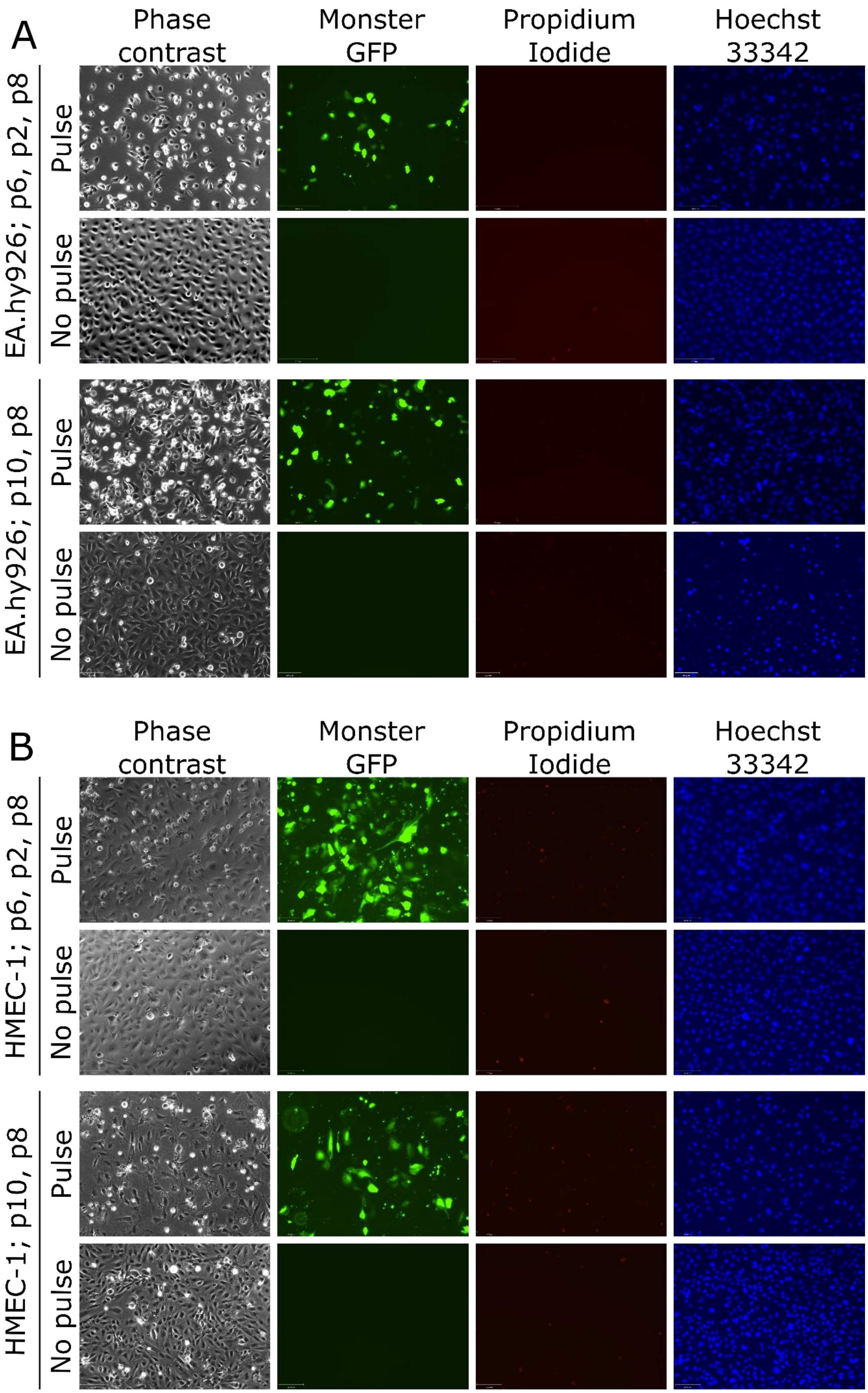
A
Phase contrast
Monster GFP
Propidium Iodide
Hoechst 33342
EA.hy926; p6, p2, p8
Pulse
No pulse
EA.hy926; p10, p8
Pulse
No pulse
B
Phase contrast
Monster GFP
Propidium Iodide
Hoechst 33342
HMEC-1; p6, p2, p8
Pulse
No pulse
HMEC-1; p10, p8
Pulse
No pulse

Fig. S5. Evaluation of transfection efficiencies by transfecting with gene network analogous plasmids, with the tTA depended reporter plasmid being replaced by a constitutive GFP expressing plasmid. Phase contrast (showing cell morphologies), green fluorescent (showing constitutive monster GFP expression from transfected p8), red fluorescent (indicating cell death by propidium iodide staining) and blue fluorescent (exhibiting the nucleus of all cells by Hoechst 33342 staining) images were recorder at 10X magnification. (A) Microscopy images of EA.hy926 endothelial cells electroporated with two different sets of plasmids: (1) p6, p2, p8 and (2) p10, p8. Images of cells to which these plasmids were added, but were not pulsed are also shown as negative controls. The optimal pulse for EA.hy926 cells was 800 V, 30 ms, double pulse. (B) Microscopy images of HMEC-1 endothelial cells electroporated with two different sets of plasmids: (1) p6, p2, p8 and (2) p10, p8. Images of cells to which these plasmids were added, but were not pulsed are also shown as negative controls. The optimal pulse for HMEC-1 cells was 1200 V, 30 ms, double pulse. For additional details on plasmids cf. Table S1 and Fig. S3 below. For details on transfection efficiencies when electroporating with these plasmid sets, see Fig S4 below.

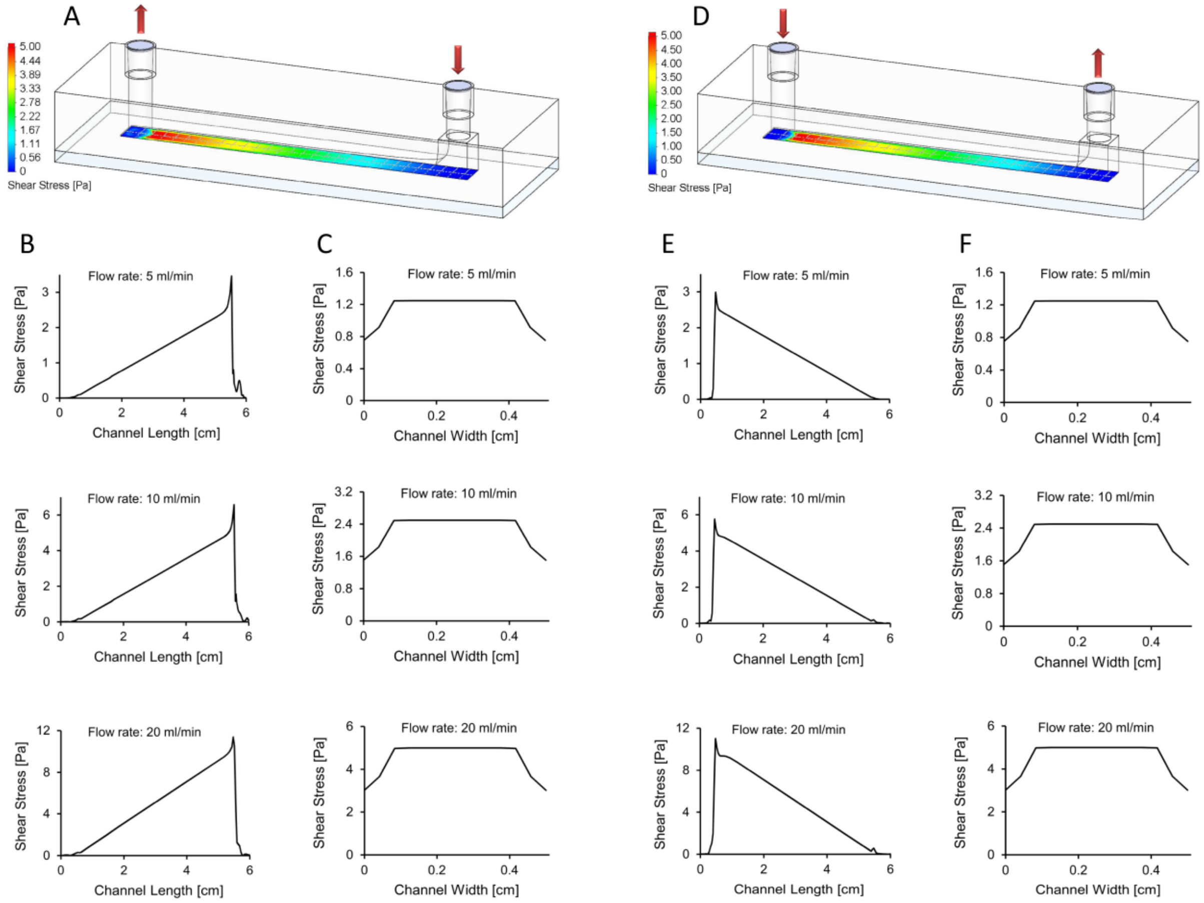

Fig. S6. Design and computational validation of the in-house designed linear shear stress inducing flow chamber. (A) Geometry of the bespoke height-variance flow channel with shear stress map at the channel floor, where cells can be seeded. The flow entered with a flow rate of 10 ml/min at the high height end of the channel and the flow exited at the low height end of the channel. (B) Evolution of shear stress along the length of the channel floor, in the width-wise centre of the channel floor, at 3 flow rates (5, 10, 20 ml/min). (C) Shear stress plotted across the width of the channel floor, in the length-wise centre of the channel floor, at 3 flow rates. (D) Geometry of the bespoke height-variance flow channel with reverse flow direction shear stress map at the channel floor, where cell seeding takes place. The

flow entered with a flow rate of 10 ml/min at the low height end of the channel and exited at the high height end of the channel. (E) Shear stress along the length of the channel floor, in the width-wise centre of the channel floor, at 3 flow rates, with flow entering at the low height inlet of the channel. (F) Shear stress computed across the width of the channel floor, in the length-wise centre of the channel floor, at 3 flow rates, with flow entering at the low height inlet of the channel.

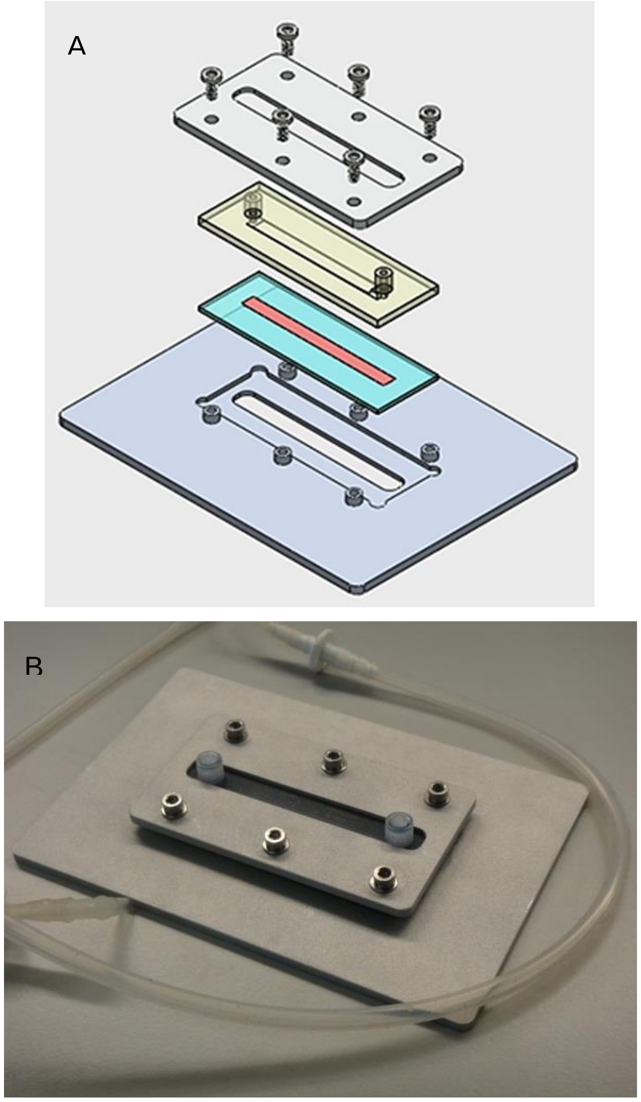

Fig. S7. Complementary equipment and the assembly of the flow device. (A) Illustration of the device parts. From the bottom: microscope holder and bottom clamp plate; microscopy glass slide on which cell are seeded and which constitutes the bottom of the flow channel; bottomless flow channel; top clamp plate; screws which fit into the bottom plate. (B) Image of the manufactured and assembled flow device.

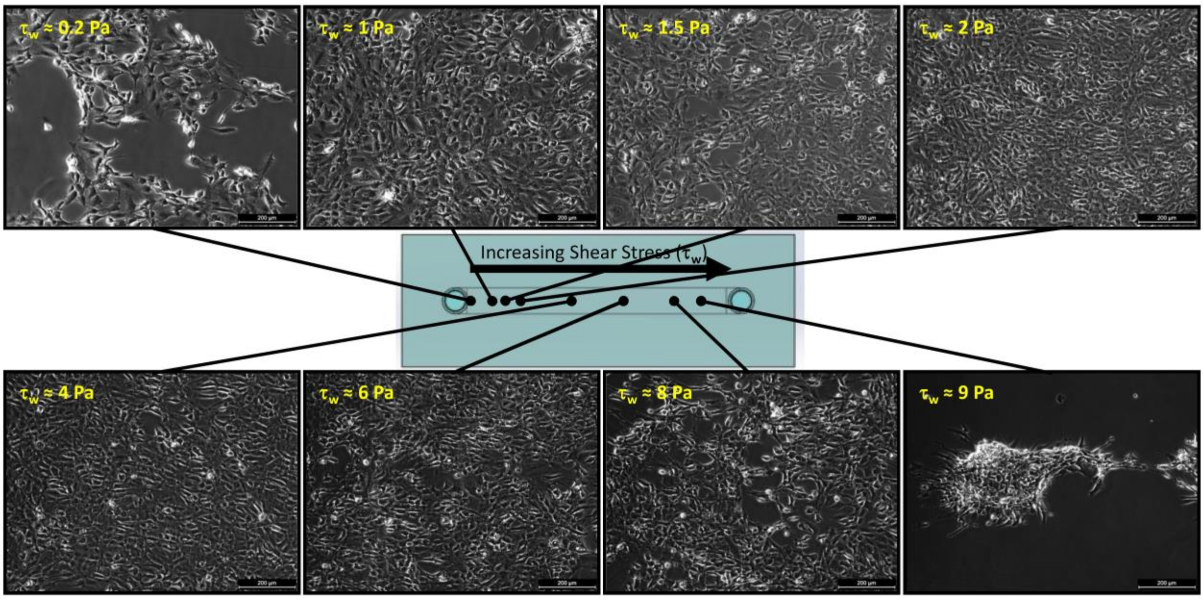

Fig. S8. Validation of the in-house developed linear shear stress inducing flow setup by flow experiments on mammalian cells in the 0–10 Pa range. Phase contrast microscopic images of primary porcine aortic endothelial cells exposed to linearly increasing shear stress along the length of the channel floor in our in-house designed flow device. Cells were seeded in the PDMS seeding well coated with 1 mg/ml fibronectin. 24 hours after seeding, the PDMS seeding well was detached from the glass slide and was replaced with the PDMS bottomless flow channel. Next, the flow experiment was started with lower flow rate in the initial two hours and maintained at 0–10 Pa yielding flow rate thereafter for 24 hours. The presence of cells and flow alignment starting above 1.5 Pa confirmed the success of our in-house designed channel. 10X magnification. N=3.

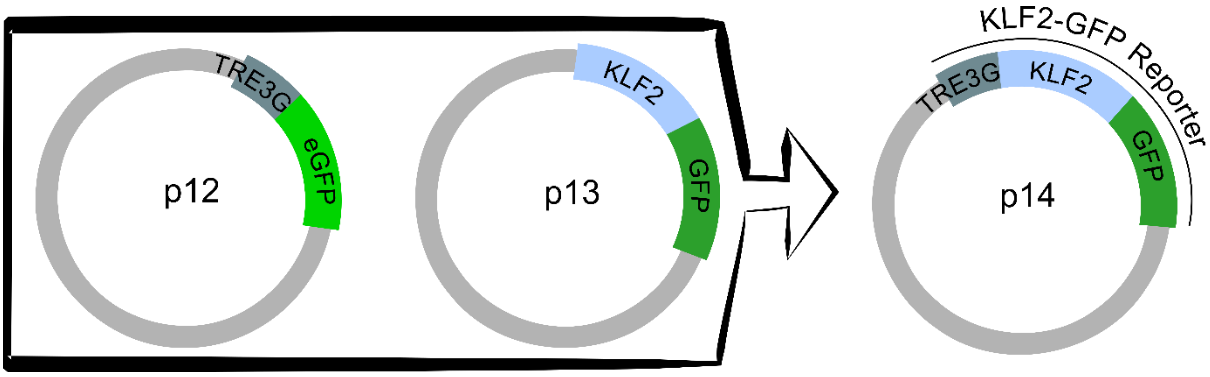

Fig. S9. Simplified illustration of plasmids elements used for building the KFL2-GFP reporter plasmids. The KLF2-GFP fusion gene from p13 replaced the eGFP gene from p12. This way plasmid 14 was created which contains the p12 backbone, the 3rd generation tetracycline responsive promoter (TRE3G) and the human Krüppel-like Factor 2 (KLF2) gene fused to the green fluorescent protein gene (GFP). For additional details regarding plasmids cf. Table S1.

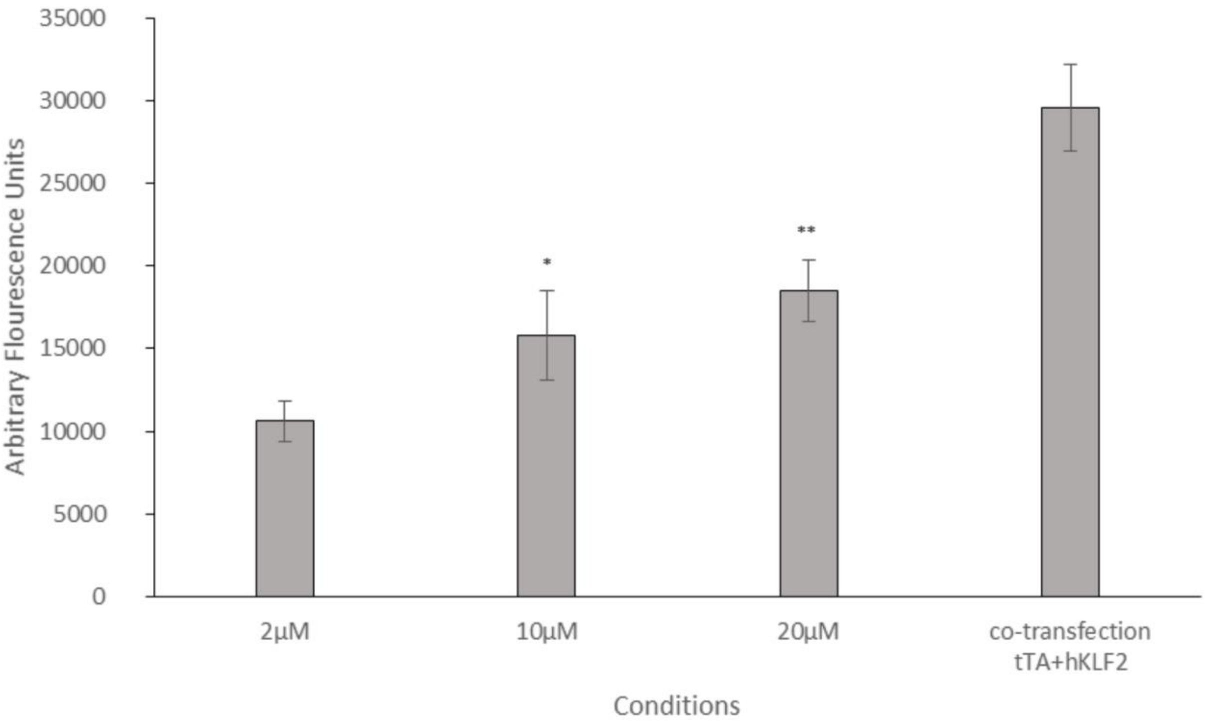

Fig. S10. Activation of the KLF2 modulator gene network by increasing bradykinin concentrations. HMEC-1 cells were electroporated with p10, selected with neomycin for 48 hours, electroporated with p14 and induced 2, 10 and 20 µM final concentrations of bradykinin. Twenty-four hours after bradykinin induction cells were stained with 2 µg/ml Hoechst 33342 and 0.5 µg/ml propidium iodide and then fixed with 4% (v/v) paraformaldehyde. Fixed cell were imaged by fluorescent microscopy. Single cells from recorded images were analyzed to determine fluorescence intensities (due to KLF2-GFP expression) at each bradykinin concentration. As a positive control, HMEC-1 cells were also co-transfected with a constitutive tTA expressing plasmid and p14 without bradykinin addition. Error bars represent standard deviation. N=10 (single cells analyzed), * $P<0.001$ for 2 µM vs. 10 µM condition, ** $P<0.001$ for 2 µM vs. 20 µM condition. Statistical significance was calculated using two way ANOVA.

References:

1. Birnboim, H.C. & Doly, J. A rapid alkaline extraction procedure for screening recombinant plasmid DNA. *Nucleic Acids Res* **7**, 1513-1523 (1979).
2. Thermo Fisher Scientific Inc. Assessment of Nucleic Acid Purity, NanoDrop Spectrophotometers, T042-TECHNICAL BULLETIN Thermo Fisher Scientific Inc. Cited: 19.Feb.2013. Available from: http://www.nanodrop.com/Library/T042-NanoDrop-Spectrophotometers-Nucleic-Acid-Purity-Ratios.pdf.
3. Magrane, M. & Consortium, U. UniProt Knowledgebase: a hub of integrated protein data. *Database (Oxford)* **2011**, bar009 (2011).
4. (NCBI), N.C.f.B.I. NCBI Protein Database. (U.S. National Library of Medicine, 2015).
5. Berman, H.M., Westbrook, J., Feng, Z., Gilliland, G., *et al.* The Protein Data Bank. *Nucleic acids research* **28**, 235-242 (2000).
6. Eddy, S.R. Profile hidden Markov models. *Bioinformatics* **14**, 755-763 (1998).
7. McWilliam, H., Li, W., Uludag, M., Squizzato, S., *et al.* Analysis Tool Web Services from the EMBL-EBI. *Nucleic acids research* **41**, W597-600 (2013).
8. Sievers, F., Wilm, A., Dineen, D., Gibson, T.J., *et al.* Fast, scalable generation of high-quality protein multiple sequence alignments using Clustal Omega. *Mol Syst Biol* **7**, 539 (2011).
9. Sonnhammer, E.L., von Heijne, G. & Krogh, A. A hidden Markov model for predicting transmembrane helices in protein sequences. *Proc Int Conf Intell Syst Mol Biol* **6**, 175-182 (1998).
10. Krogh, A., Larsson, B., von Heijne, G. & Sonnhammer, E.L. Predicting transmembrane protein topology with a hidden Markov model: application to complete genomes. *J Mol Biol* **305**, 567-580 (2001).
11. Kall, L., Krogh, A. & Sonnhammer, E.L. A combined transmembrane topology and signal peptide prediction method. *J Mol Biol* **338**, 1027-1036 (2004).
12. Kall, L., Krogh, A. & Sonnhammer, E.L. Advantages of combined transmembrane topology and signal peptide prediction--the Phobius web server. *Nucleic acids research* **35**, W429-432 (2007).
13. Kelley, L.A. & Sternberg, M.J. Protein structure prediction on the Web: a case study using the Phyre server. *Nat Protoc* **4**, 363-371 (2009).
14. Schrödinger, L. The PyMOL Molecular Graphics System, Version 1.5.0.4. (PyMOL, 2014).
15. New England BioLabs Inc. Tm Calculator. 2013. Cited: 12. Sep. 2013. Version: 1.0.0.3798. Available from: https://www.neb.com/tools-and-resources/interactive-tools/tm-calculator.
16. SantaLucia, J., Jr. A unified view of polymer, dumbbell, and oligonucleotide DNA nearest-neighbor thermodynamics. *Proceedings of the National Academy of Sciences of the United States of America* **95**, 1460-1465 (1998).
17. Owczarzy, R., You, Y., Moreira, B.G., Manthey, J.A., *et al.* Effects of sodium ions on DNA duplex oligomers: Improved predictions of melting temperatures. *Biochemistry-Us* **43**, 3537-3554 (2004).
18. Breslauer, K.J., Frank, R., Blocker, H. & Marky, L.A. Predicting DNA duplex stability from the base sequence. *Proc Natl Acad Sci U S A* **83**, 3746-3750 (1986).

19. Vogelstein, B. & Gillespie, D. Preparative and analytical purification of DNA from agarose. *Proc Natl Acad Sci U S A* **76**, 615-619 (1979).

20. Blaukat, A., Barac, A., Cross, M.J., Offermanns, S.*, et al.* G protein-coupled receptor-mediated mitogen-activated protein kinase activation through cooperation of Galpha(q) and Galpha(i) signals. *Molecular and cellular biology* **20**, 6837-6848 (2000).

21. McEachern, A.E., Shelton, E.R., Bhakta, S., Obernolte, R.*, et al.* Expression cloning of a rat B2 bradykinin receptor. *Proc Natl Acad Sci U S A* **88**, 7724-7728 (1991).

22. Blaukat, A., Alla, S.A., Lohse, M.J. & Muller-Esterl, W. Ligand-induced phosphorylation/dephosphorylation of the endogenous bradykinin B2 receptor from human fibroblasts. *The Journal of biological chemistry* **271**, 32366-32374 (1996).

23. Schanstra, J.P., Neau, E., Drogoz, P., Arevalo Gomez, M.A.*, et al.* In vivo bradykinin B2 receptor activation reduces renal fibrosis. *The Journal of clinical investigation* **110**, 371-379 (2002).

24. Decarie, A., Raymond, P., Gervais, N., Couture, R.*, et al.* Serum interspecies differences in metabolic pathways of bradykinin and [des-Arg(9)]BK: Influence of enalaprilat. *Am J Physiol-Heart C* **271**, H1340-H1347 (1996).

25. Shima, C., Majima, M. & Katori, M. A Stable Metabolite, Arg-Pro-Pro-Gly-Phe, of Bradykinin in the Degradation Pathway in Human Plasma. *Jpn J Pharmacol* **60**, 111-119 (1992).

26. Murphey, L.J., Hachey, D.L., Oates, J.A., Morrow, J.D.*, et al.* Metabolism of bradykinin in vivo in humans: Identification of BK1-5 as a stable plasma peptide metabolite. *J Pharmacol Exp Ther* **294**, 263-269 (2000).

27. Tom, B., Dendorfer, A., de Vries, R., Saxena, P.R.*, et al.* Bradykinin potentiation by ACE inhibitors: a matter of metabolism. *Brit J Pharmacol* **137**, 276-284 (2002).

28. Ferreira, S.H. & Vane, J.R. The disappearance of bradykinin and eledoisin in the circulation and vascular beds of the cat. *Br J Pharmacol Chemother* **30**, 417-424 (1967).

29. Feletou, M., Germain, M., Thurieau, C., Fauchere, J.L.*, et al.* Agonistic and antagonistic properties of the bradykinin B2 receptor antagonist, Hoe 140, in isolated blood vessels from different species. *Br J Pharmacol* **112**, 683-689 (1994).

30. Wirth, K., Hock, F.J., Albus, U., Linz, W.*, et al.* Hoe 140 a new potent and long acting bradykinin-antagonist: in vivo studies. *Br J Pharmacol* **102**, 774-777 (1991).

31. Hock, F.J., Wirth, K., Albus, U., Linz, W.*, et al.* Hoe 140 a new potent and long acting bradykinin-antagonist: in vitro studies. *Br J Pharmacol* **102**, 769-773 (1991).

32. Yu, J., Zhang, L., Hwang, P.M., Rago, C.*, et al.* Identification and classification of p53-regulated genes. *Proc Natl Acad Sci U S A* **96**, 14517-14522 (1999).

33. Zhu, P., Aller, M.I., Baron, U., Cambridge, S.*, et al.* Silencing and un-silencing of tetracycline-controlled genes in neurons. *PLoS One* **2**, e533 (2007).

34. Rasband, W.S. ImageJ. Bethesda, Maryland. U. S. National Institutes of Health. 1997-2014. Cited: 2014 11.Feb.2014. Available from: http://imagej.nih.gov/ij/.

35. Schneider, C.A., Rasband, W.S. & Eliceiri, K.W. NIH Image to ImageJ: 25 years of image analysis. *Nature methods* **9**, 671-675 (2012).

36. Chachisvilis, M., Zhang, Y.L. & Frangos, J.A. G protein-coupled receptors sense fluid shear stress in endothelial cells. *Proc Natl Acad Sci U S A* **103**, 15463-15468 (2006).

37. Makino, A., Prossnitz, E.R., Bunemann, M., Wang, J.M., *et al.* G protein-coupled receptors serve as mechanosensors for fluid shear stress in neutrophils. *Am J Physiol Cell Physiol* **290**, C1633-1639 (2006).

38. Zhang, Y.-L., Frangos, J.A. & Chachisvilis, M. Mechanical stimulus alters conformation of type 1 parathyroid hormone receptor in bone cells. *Am J Physiol Cell Physiol* **296**, C1391-1399 (2009).

39. Abdul-Majeed, S. & Nauli, S.M. Dopamine receptor type 5 in the primary cilia has dual chemo- and mechano-sensory roles. *Hypertension* **58**, 325-331 (2011).

40. Jung, B., Obinata, H., Galvani, S., Mendelson, K., *et al.* Flow-regulated endothelial S1P receptor-1 signaling sustains vascular development. *Dev Cell* **23**, 600-610 (2012).

41. Takada, Y., Kato, C., Kondo, S., Korenaga, R., *et al.* Cloning of cDNAs encoding G protein-coupled receptor expressed in human endothelial cells exposed to fluid shear stress. *Biochem Biophys Res Commun* **240**, 737-741 (1997).

42. Meyer, M.R., Fredette, N.C., Howard, T.A., Hu, C., *et al.* G protein-coupled estrogen receptor protects from atherosclerosis. *Sci Rep* **4**, 7564 (2014).
